# Mechanism‐Informed Machine Learning Enables Discovery of Oncolytic Peptides for Cancer Immunotherapy

**DOI:** 10.1002/advs.75652

**Published:** 2026-05-12

**Authors:** Wen Zhang, Shengxin Lu, Guangyong Zheng, Shensuo Li, Hongyu Chen, Mei Hong, Xiangru Zhou, Ruotian Tang, Ye Wu, Weidong Zhang, Dong Lu, Xin Luan

**Affiliations:** ^1^ State Key Laboratory of Discovery and Utilization of Functional Components in Traditional Chinese Medicine Shanghai Frontiers Science Center of Chinese Medicine Chemical Biology Institute of Interdisciplinary Integrative Medicine Research and Shuguang Hospital Shanghai University of Traditional Chinese Medicine Shanghai China; ^2^ West China School of Public Health and West China Fourth Hospital State Key Laboratory of Biotherapy Sichuan University Chengdu China; ^3^ Shanghai Institute of Infectious Diseases and Biosafety Institute of Interdisciplinary Integrative Medicine Research Shanghai University of Traditional Chinese Medicine Shanghai China; ^4^ School of Pharmacy Naval Medical University Shanghai China; ^5^ State Key Laboratory for Quality Ensurance and Sustainable Use of Dao‐di Herbs Institute of Medicinal Plant Development Chinese Academy of Medical Sciences and Peking Union Medical College Beijing China

**Keywords:** cancer immunotherapy, high‐confidence ensemble model, machine learning, mechanism‐informed screening pipeline, oncolytic peptide

## Abstract

Oncolytic peptides (OPs) represent a promising class of cancer therapeutics capable of rapidly lysing tumor cells and activating antitumor immunity. However, accurate in silico identification of potent OPs remains challenging due to limited datasets and high false‐positive rates. Here, we present MISPOP (Mechanism‐Informed Screening Pipeline for Oncolytic Peptides), an integrated machine learning framework that combines eXtreme Gradient Boosting, deep neural networks, and transfer learning into a high‐confidence ensemble model augmented with physicochemical priors. Applied to a natural peptide library of 1033 sequences, MISPOP prioritized 16 candidates, among which five were synthesized and evaluated across three tumor cell lines. Dermaseptin‐S9 exhibited the most favorable therapeutic index. Molecular dynamics simulations revealed its deep insertion into lipid bilayers and stable peptide‐membrane interactions, while in vitro assays confirmed pronounced membrane disruption and induction of immunogenic cell death. In a B16F10 melanoma model, Dermaseptin‐S9 achieved over 92% tumor growth inhibition without evident systemic toxicity. Collectively, these findings demonstrate that embedding biochemical priors into ensemble learning can markedly improve predictive accuracy and enable the discovery of potent OPs, offering a generalizable paradigm for accelerating peptide‐based oncotherapy development.

## Graphical abstract

MISPOP integrates ensemble learning with membrane‐active physicochemical priors to identify Dermaseptin‐S9, a natural oncolytic peptide that disrupts tumor membranes, triggers immunogenic cell death, and shows strong antitumor activity. The study illustrates a mechanism‐informed route from peptide sequence data to cancer immunotherapy leads.

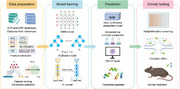

## 1 Introduction

Therapeutic peptides occupy a middle ground between small molecules and biologics, combining high selectivity, low immunogenicity, tunable chemistry, favorable tissue penetration, and scalable manufacture [1, 2]. As of 2024, over 120 peptide drugs have been approved worldwide, most originating from natural scaffolds or optimized analogues. Their approvals have risen steadily over the past six decades, paralleling a global market expanding by approximately 7.7% annually [3]. Consistently, a bibliometric analysis from 2005 to 2024 revealed sustained publication growth, with a marked acceleration following the approval of oral semaglutide in 2019 [4]. Notably, since 2020, “cancer” has surpassed type 2 diabetes as the leading disease focus in peptide research [5], highlighting peptides as a rapidly maturing therapeutic modality.

Most approved peptides trace their origins to natural scaffolds; consequently, discovery has traditionally relied on extraction and fractionation of crude biological materials, followed by iterative purification, resynthesis, and multi‐round phenotypic screening [6]. This process is laborious, low‐throughput, and costly, often hindered by high attrition and limited guidance from bioactivity cues. Advances in artificial intelligence (AI) and machine learning (ML) have introduced a paradigm shift by enabling activity prediction directly from sequence, prioritizing testable leads, and triaging libraries in silico [7, 8]. For example, PQ203, a peptide‐drug conjugate developed through a physics‐based and generative ML platform, has advanced to a first‐in‐human phase I trial with U.S. Food and Drug Administration (FDA) Fast Track designation [9]. The first patient has already been dosed, reinforcing the feasibility of AI‐driven peptide drug discovery and strengthening confidence that computational design can shorten the path from natural reservoirs to clinic‐ready peptide therapeutics.

Within this emerging landscape, oncolytic peptides (OLPs) represent a distinct class of short, cationic amphiphiles derived mainly from natural products and endowed with broad antitumor activity [10, 11]. Mechanistically, OLPs exert their effects primarily through direct tumor‐membrane lysis while triggering immunogenic cell death (ICD), thereby converting dying tumor cells into an in situ vaccine [12, 13, 14]. This dual mechanism can bypass resistance that limits chemotherapy and targeted agents and functions independently of pre‐existing immunity required for checkpoint blockade [15]. Four OLPs, including LTX‐315 (VP‐315), CyPep‐1, EP‐100, and LL‐37, are in clinical testing, underscoring translational momentum [16]. Among them, LTX‐315 has completed multiple trials with encouraging outcomes [17, 18]. In an ongoing phase II trial for basal cell carcinoma (ClinicalTrials.gov NCT05188729), LTX‐315 induced lesion shrinkage in 86% of treated tumors and complete histologic clearance in more than half, without treatment‐related serious adverse events, highlighting both efficacy and tolerability. These findings position OLPs as a mechanistically distinct modality with strong potential to overcome therapeutic resistance while mobilizing durable antitumor immunity.

OLPs largely originate from natural peptides, particularly antimicrobial peptides (AMPs) from venoms, skin secretions, and innate immune effectors. Our prior studies characterized anoplin [19, 20], mastoparan [21, 22], and tachyplesin I [23, 24] as natural OLPs. However, their discovery still depends on extraction or chemical synthesis followed by phenotypic screening, which remains time‐consuming, low‐throughput, and resource‐intensive [25, 26]. Concurrently, several computational tools have been developed for AMP or anticancer peptide (ACP) identification, leveraging support vector machines or deep‐learning architectures trained on amino‐acid composition, binary sequence profiles, and physicochemical descriptors [27, 28, 29]. Nevertheless, these models are optimized for broad antibacterial or antiproliferative activity rather than the oncolytic phenotype. Consequently, they fail to capture key biophysical hallmarks of OLPs, such as tumor‐selective membrane disruption and ICD induction, and exhibit limited capability to prioritize peptides with genuine oncolytic potential. Moreover, the scarcity of curated OLP‐specific datasets restricts model training and contributes to high false‐positive rates during experimental follow‐up. These limitations underscore the need for a mechanism‐informed predictive strategy that integrates membrane‐active priors to yield high‐confidence OLP candidates for downstream validation.

**FIGURE 1:**
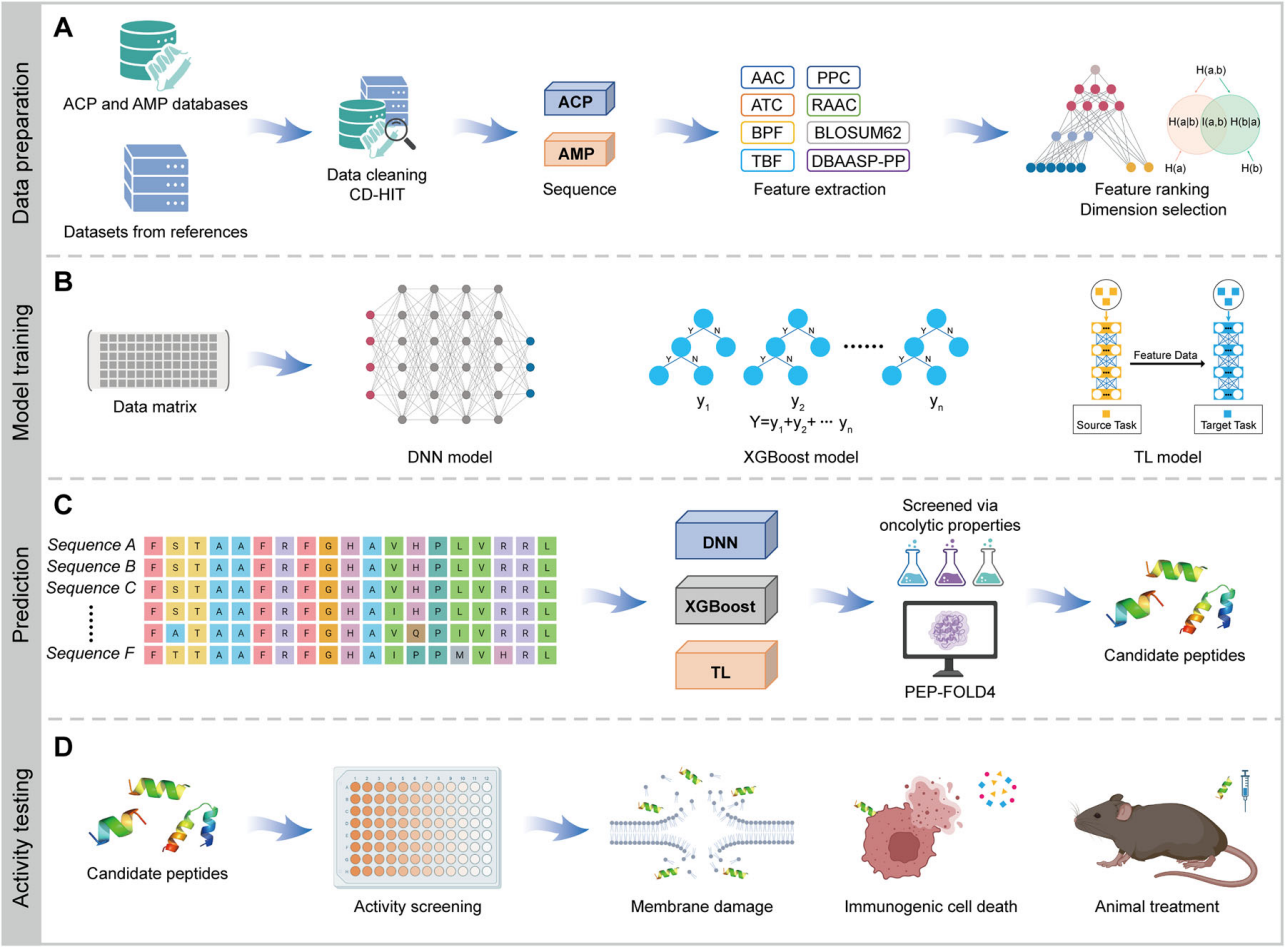
Workflow for the discovery of new OLPs. (A) Collection and preprocessing of peptide sequences, followed by feature extraction and dimensionality reduction. (B) Construction of ACP prediction models using ML. (C) High‐confidence screening strategy for identifying candidate OLPs. (D) Experimental validation of candidate ACPs, including in vitro antitumor activity assays, mechanistic evaluation of oncolysis, and in vivo therapeutic efficacy studies.

To address the limited availability of ACP data and the paucity of curated OLP labels, we incorporated AMP sequences and the characteristic biophysical parameters of OLPs into our screening strategy. We compiled training datasets from established ACP and AMP repositories, extracted multi‐dimensional sequence and physicochemical features, and applied dimensionality reduction (Figure 1A) to train three independent models based on eXtreme Gradient Boosting (XGBoost), deep neural networks (DNN), and transfer learning (TL, Figure 1B) [30, 31, 32]. We then developed a two‐stage screening framework, called MISPOP (**M**echanism‐**I**nformed **S**creening **P**ipeline for **O**ncolytic **P**eptides), for OLP recognition (Figure 1C). In stage I, MISPOP performs high‐confidence consensus screening across the three predictors to enrich for ACP‐like sequences. In stage II, it integrates amphiphilicity, hydrophobicity, and net charge with secondary‐structure prediction to favor helical, membrane‐active OLP candidates. We prospectively applied MISPOP to natural peptide libraries and subsequently validated the resulting leads through molecular‐dynamics simulations and biological assays to connect sequence‐level features with membrane engagement and ICD induction (Figure 1D). This mechanism‐informed discovery route bridges computational prediction with experimental validation, reduces screening burden, and establishes a generalizable paradigm for accelerating OLP discovery for cancer immunotherapy.

## 2 Results and Discussion

### 2.1 Establishment of the Basic Prediction Models

**FIGURE 2:**
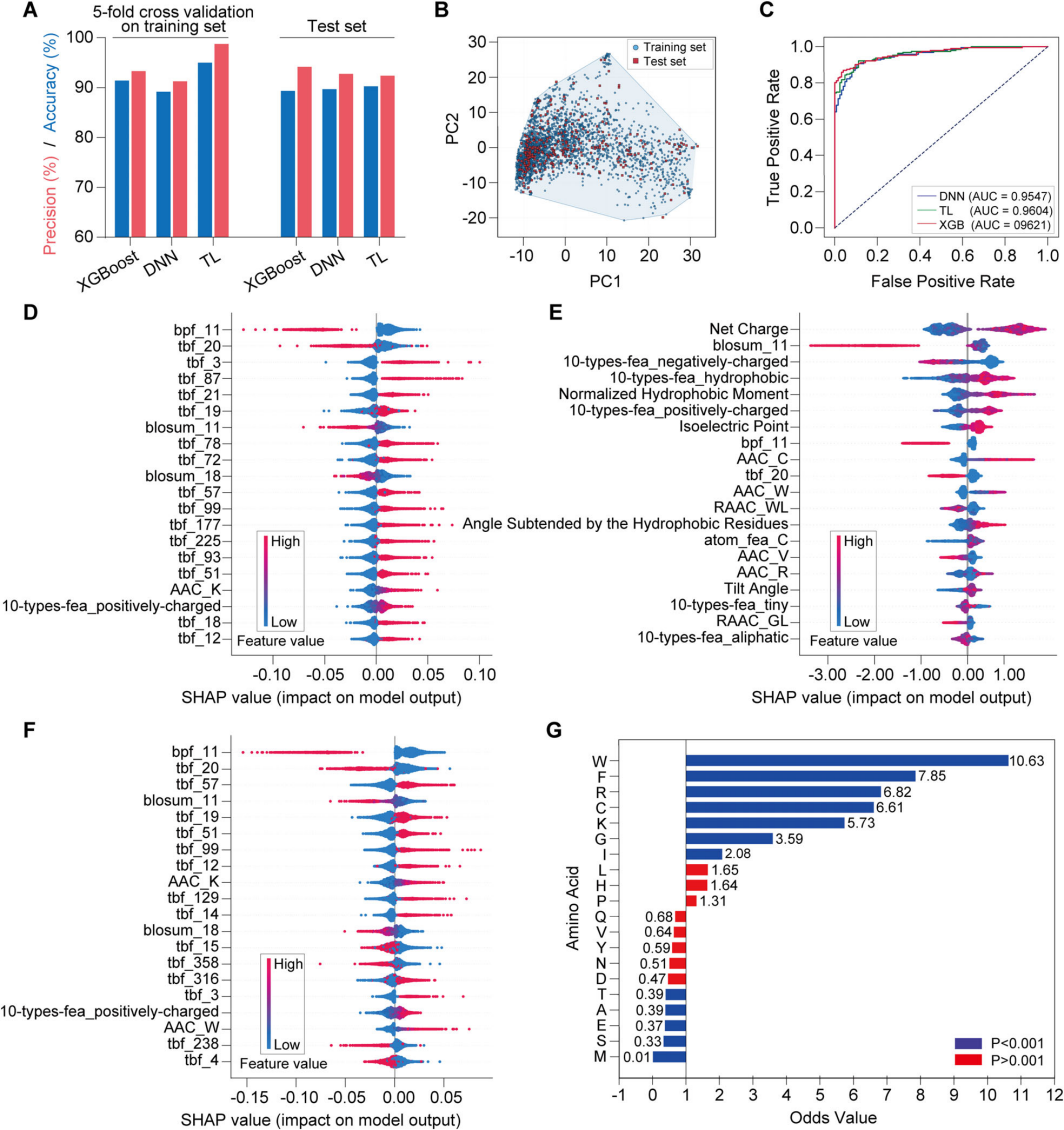
Construction and evaluation of the ML models. (A) Comparison of accuracy and precision among the XGBoost, DNN, and TL models on the training and test sets. (B) Principal component analysis of the training and test datasets. (C) ROC curve of the TL, DNN, and XGBoost models evaluated on the test set. D‐F, SHAP analysis of the (D) TL, (E) XGBoost, and (F) DNN models, respectively. The y‐axis shows the top 20 features contributing to each model, while the x‐axis indicates their corresponding SHAP values. Dot color represents the feature value of each sample (blue: low, red: high). (G) Fisher's exact test comparing the first amino acid residues in ACP and non‐ACP sequences. The x‐axis indicates the odds ratio, while the y‐axis lists the amino acids. Bar color denotes statistical significance (blue: *P* < 0.001; red: *P* > 0.001).

We curated a nonredundant dataset consisting of 1921 experimentally validated ACPs as positive samples and 1253 negative sequences that were not annotated as anticancer or antimicrobial peptides after database filtering and curation. The dataset was then split into training and test sets. Sequence features were calculated to generate numerical representations. To reduce feature redundancy and mitigate overfitting, we applied the minimum Redundancy Maximum Relevance (mRMR) algorithm for feature selection. On this basis, three basic prediction models were developed using TL, DNN, and XGBoost. Model performance was evaluated by fivefold cross‐validation on the training set and by independent testing on the test set (Table 1 and Figure 2A). All three models achieved high accuracy and precision, each exceeding 85% on both the training and test sets. A principal component analysis showed that the training distribution covered 99.05% of the test set, indicating that the test samples could be well predicted within the application domain of the model (Figure 2B). Receiver operating characteristic (ROC) curves further demonstrated excellent discrimination between positive and negative samples (Figure 2C).

**TABLE 1:** Performance of the XGBoost, DNN, and TL models. Acc: accuracy; Pre: precision; Sen: sensitivity; Spe: specificity; MCC: Matthews Correlation Coefficient; AUC: area under the ROC curve.

Models	Acc	Pre	Sen	Spe	MCC	AUC
fivefold cross‐validation on the training set						
XGBoost	0.9167	0.9357	0.9260	0.9025	0.8264	0.9698
DNN	0.8943	0.9151	0.9098	0.8706	0.7794	0.9623
TL	0.9524	0.9901	0.9306	0.9858	0.9046	0.9868
Test set						
XGBoost	0.8959	0.9441	0.8802	0.9200	0.7888	0.9621
DNN	0.8991	0.9301	0.9010	0.8960	0.7910	0.9547
TL	0.9054	0.9263	0.9167	0.8880	0.8025	0.9604

### 2.2 SHAP Analysis of the Models

We used SHAP (Shapley Additive Explanations) analysis to quantify feature contributions to each predictor [33]. For all three models, the top 20 features were identified (Figure 2D‐F). Positive SHAP values indicate a shift toward ACP classification, whereas negative values favor non‐ACP. For example, “bpf_11” ranked first in both the DNN and TL models; its values were low in ACPs and high in non‐ACPs, so a peptide with a high “bpf_11” value is likely a non‐ACP. Four features were consistently important across TL, DNN, and XGBoost: “bpf_11”, “tbf_20”, “blosum_11”, and “10‐types‐fea_positively‐charged”. Here, “bpf_11” encodes whether methionine is present at position 1 (1 = present, 0 = absent). “tbf_20” denotes whether the first residue belongs to the solvent‐accessibility group (H, M, P, S, T, Y). “blosum_11” (BLOSUM62 encoding) reflects the propensity for the first residue to be replaced by methionine. Together, these features indicate that ACP sequences tend not to start with methionine or with residues in that solvent‐accessibility group. Additional position‐1 associated features were also enriched among the top contributors (for example, “tbf_3”, “tbf_21”, “tbf_18”, “tbf_12”, “blosum_18”, “tbf_19”, “tbf_14”, “tbf_15”, and “tbf_4”). Consistently, Fisher's exact test showed significant differences in the N‐terminal residue between ACPs and non‐ACPs (Figure 2G; Table S6). W, F, R, C, K, G, and I were enriched in ACPs (odds ratio > 1), whereas T, A, E, S, and M were more frequent in non‐ACPs (P < 0.001). In addition, a higher value of “10‐types‐fea_positively‐charged” suggests a greater probability of being an ACP.

XGBoost mainly prioritized global physicochemical and composition features, unlike TL and DNN (Figure 2E). Net charge was the top feature, implying that a higher positive charge increases the probability of being an ACP. Lower values of “10‐types‐fea_negatively‐charged” are likewise associated with ACPs, reinforcing the role of electropositivity. Hydrophobicity‐related descriptors (e.g., “10‐types‐fea_hydrophobic” and “10‐types‐fea_aliphatic”) were also prominent, in agreement with the established contribution of hydrophobicity and amphiphilicity to peptide‐membrane interactions and anticancer effects. In summary, amphiphilicity, hydrophobicity, and positive charge are critical determinants of ACP activity. Notably, the top 20 features prioritized by XGBoost were mostly distinct from those of TL and DNN. The DNN and TL models shared 12 features, while the remaining 8 features were unique to each model. Differences in top features across the three models indicate complementary learned representations and support the value of high‐confidence ensemble models.

### 2.3 Ensemble Models With High Confidence

**FIGURE 3:**
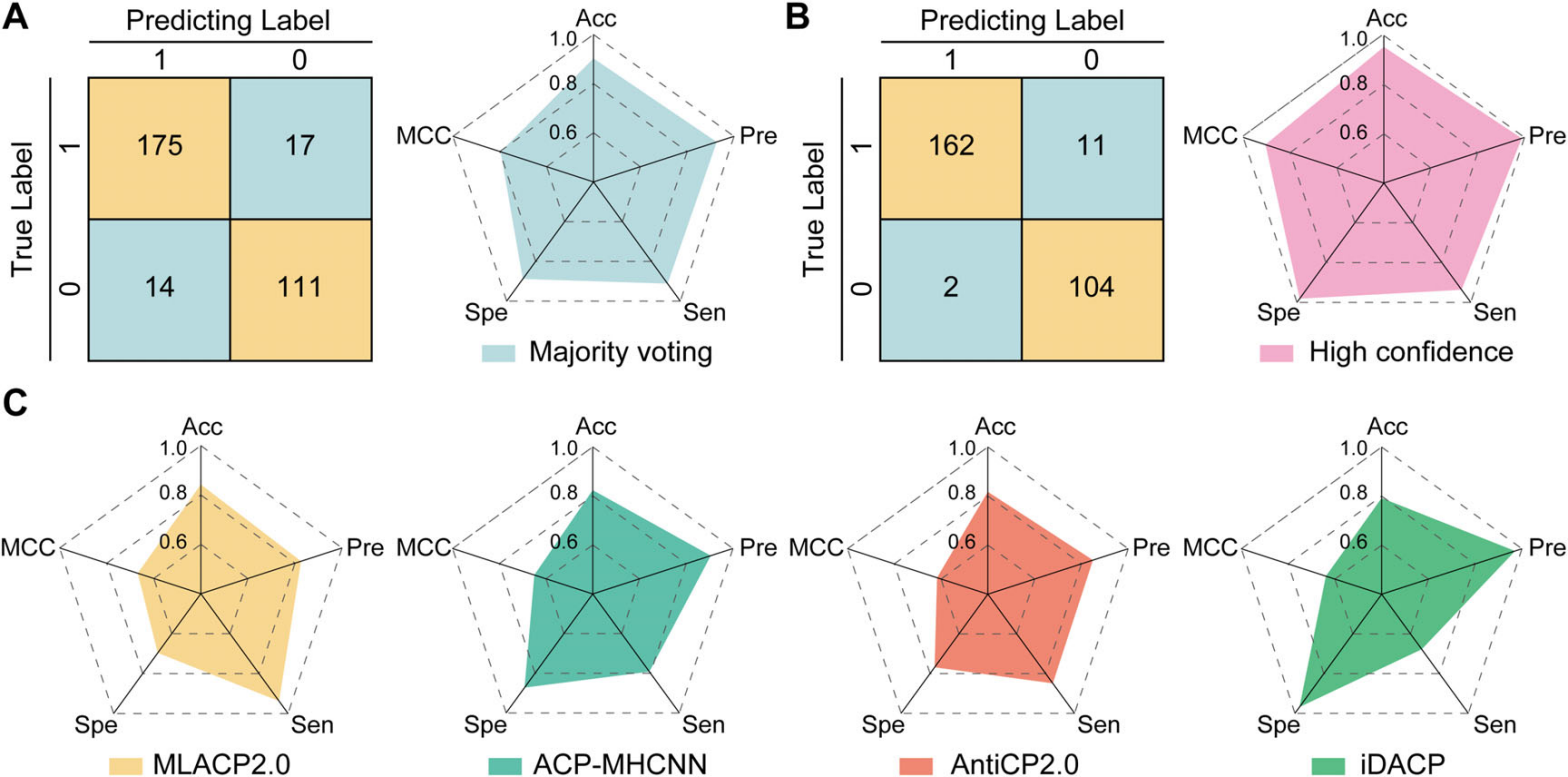
Performance evaluation of ML models. (A, B) Confusion matrices and performance scores of sample predictions generated by the (A) majority voting model and the (B) high‐confidence model. (C) Comparison of classification metrics of a previously published ACP prediction model evaluated on the same test set.

To improve ACP recognition, we integrated the outputs of the three base learners. We compared a majority voting model, which accepts predictions agreed by at least two models, with a high confidence model (HCM), which requires unanimous agreement across all three. On the test set, HCM markedly reduced false positives compared with majority voting (1.22% vs 7.41%), as shown by the confusion matrices in Figure 3A,B, and outperformed any single model (Table 2). Because HCM filters out samples with discordant predictions, it trades a small loss in coverage for a substantial gain in reliability, which is desirable for prospective screening where experimental validation is costly. Accordingly, HCM was adopted as the stage‐one consensus model in MISPOP, providing a high‐confidence gate that lowers downstream experimental burden while maintaining robust discrimination.

**TABLE 2:** Performance comparison of the established models and previously published ACP prediction methods on the test set.

Type	Models	Acc	Pre	Sen	Spe	MCC	AUC
This study							
Individual model	XGBoost	0.8959	0.9441	0.8802	0.9200	0.7888	0.9621
	DNN	0.8991	0.9301	0.9010	0.8960	0.7910	0.9547
	TL	0.9054	0.9263	0.9167	0.8880	0.8025	0.9604
Combining model	Majority voting	0.9022	0.9259	0.9115	0.8880	0.7963	—
	High confidence	0.9534	0.9878	0.9364	0.9811	0.9048	—
Comparative methods							
	ACP‐MHCNN [34]	0.8233	0.9048	0.7917	0.8720	0.6498	—
	ACPred‐BMF [35]	0.8076	0.8394	0.8438	0.7520	0.5966	—
	AntiCP 2.0 [36]	0.8170	0.8490	0.8490	0.7680	0.6170	0.9085
	iDACP [37]	0.7918	0.9701	0.6771	0.9680	0.6382	0.9403
	iACP‐FSCM [38]	0.6215	0.7813	0.5208	0.7760	0.2957	—
	ACP‐MLC [39]	0.7319	0.7956	0.7500	0.7040	0.4483	0.8061
	ACP‐Net [40]	0.5458	0.5920	0.8000	0.1570	−0.0543	0.4743
	DLFF‐ACP [41]	0.7003	0.7904	0.6875	0.7200	0.3989	0.7966
	MLACP 2.0 [42]	0.8423	0.8257	0.9375	0.6960	0.6680	0.9456
	acp‐ope [43]	0.6719	0.7716	0.6510	0.7040	0.3471	0.7630

### 2.4 Performance Comparison With Other ACP Prediction Models

To contextualize our approach, we benchmarked the three base learners and the HCM against representative ACP predictors (ACP‐MHCNN, ACPred‐BMF, MLACP2.0, etc.) on the same test set (Table 2). All three base models performed competitively, and the HCM achieved the best overall balance across accuracy, precision, specificity, AUC, and MCC (Figure 3A‐C). MLACP2.0 showed slightly higher sensitivity on this dataset, but at the cost of more false positives, whereas HCM maintained a lower false positive rate and higher precision. Consequently, HCM delivered superior aggregate performance on metrics that are most relevant for prospective screening, where minimizing experimental follow‐up on false positives is critical. These results support HCM as a reliable and effective tool for identifying natural peptides with antitumor activity and justify its use as the stage one consensus in MISPOP.

### 2.5 HCM‐Guided Discovery of OLPs

**FIGURE 4:**
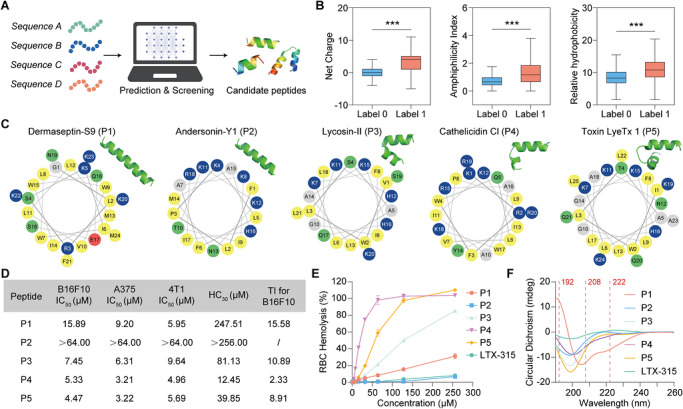
ML‐driven prediction, screening, and experimental validation of OLPs. (A) Schematic diagram of the ML‐based prediction and screening pipeline for OLPs. (B) Physicochemical features used for secondary screening of OLPs. Net charge, amphiphilic index, and relative hydrophobicity significantly differed between ACPs (label_1) and non‐ACPs (label_0). (C) Helical wheel projections of the top five predicted peptides. Yellow symbols denote highly hydrophobic residues. Gray symbols denote low hydrophobic residues. Green symbols denote polar, uncharged residues. Blue symbols denote basic residues. (D) Antiproliferative activity, hemolytic activity, and TI of candidate peptides in (C). IC_50_, peptide concentration causing 50% tumor cell growth inhibition; HC_30_, peptide concentration inducing 30% red blood cell hemolysis; TI = HC_30_/IC_50_. (E) Hemolytic activity of candidate peptides in (C). (F) CD spectra of candidate peptides in (C) and LTX‐315. Data are presented as mean ± SD, ***P < 0.001.

We next applied MISPOP to discover new OLPs (Figure 4A). Prior studies indicate that strong amphiphilicity, higher hydrophobicity, greater α‐helical content, and increased cationic charge correlate with oncolysis [44]. To improve recognition accuracy, we incorporated these physicochemical priors as auxiliary features: amphiphilicity index, relative hydrophobicity, and net charge. These features differed significantly between ACPs and non‐ACPs (Figure 4B). We further predicted secondary structure with PEPFOLD4 to enrich for α‐helical candidates [45]. Because excessive hydrophobicity and helicity can lead to hemolysis, we estimated hemolytic risk using the Lyticity Index (LI) and set an exclusion threshold of LI > 600. Peptides passing these criteria were advanced for experimental verification.

Screening candidates were retrieved from Database of Antimicrobial Activity and Structure of Peptides (DBAASP) and constrained to 4–30 residues. After removing sequences with reported antitumor activity, 1033 peptides remained. Stage one retained 416 candidates at predicted probability > 0.99. Stage two applied safety and mechanism‐informed filters: we excluded LI > 600, then required values above the 75th percentile of non‐ACPs for relative hydrophobicity (≥10.275), amphiphilicity index (≥0.98), and net charge (≥1), and set net charge ≥4 for peptides longer than 10 residues [46]. This yielded 16 peptides. We predicted 3D structures with PEPFOLD4 (Figure S1) and calculated α‐helical content (Table S7), then selected five peptides with predicted helicity >80% for synthesis. α‐Helicity is closely associated with the membrane activity of many oncolytic peptides. A stable amphipathic α‐helix spatially segregates hydrophobic and positively charged residues, thereby facilitating membrane binding, insertion, and disruption [47]. Consistently, many oncolytic peptides reported in preclinical and translational studies are α‐helical, and increased α‐helicity has been shown to enhance membrane interactions and antiproliferative activity [12]. For example, stapled derivatives of wasp venom‐derived oncolytic peptides displayed increased α‐helicity together with improved oncolytic efficacy against tumor cells [19, 21]. Therefore, among the top‐ranked candidates identified by MISPOP, we gave higher priority to peptides with higher predicted α‐helical content for downstream pharmacological validation. The five peptides originated from *Phyllomedusa sauvagei* skin (Dermaseptin‐S9), *Odorrana andersonii* skin (Andersonin‐Y1), *Lycosa singoriensis* toxin (Lycosin‐II), *Lycosa erythrognatha* toxin (Toxin LyeTx I), and *Columba livia* secretory granules (Cathelicidin CI) [48, 49, 50]. None had reported anticancer activity. Helical wheel projections (HeliQuest) confirmed α‐helical organization with comparable physicochemical profiles (Figure 4C) [51]. Among them, Dermaseptin‐S9 (S9) displayed the largest hydrophobic surface, suggesting strong membrane activity. All five were synthesized and verified for purity and molecular weight (Figures S7–S16).

Antiproliferative activity was evaluated against melanoma (B16F10, A375) and breast cancer (4T1) cells, with LTX‐315 as a positive control (Figure 4D; Figures S2, S17, and S18). Except for Andersonin‐Y1, all candidates suppressed tumor‐cell growth, in several cases exceeding LTX‐315. Given the importance of safety, we profiled hemolysis and found Andersonin‐Y1 and LTX‐315 showed the best safety, followed by S9, which induced only 30% erythrocyte lysis at 256 µM (Figure 4E). To balance efficacy and safety, we calculated a therapeutic index (TI). As shown in Figure 4D, S9 achieved the highest TI. Circular dichroism (CD) spectra showed that S9 exhibited the most pronounced α‐helical features in aqueous solution, with characteristic negative bands at 208 and 222 nm, whereas four candidates displayed weaker or less defined helical features (Figure 4F). These results indicate that secondary‐structure prediction should be interpreted together with experimental validation. Therefore, we advanced S9 as the lead candidate for further pharmacodynamic and mechanistic studies.

### 2.6 Molecular Dynamics Reveal Selective Insertion of S9 Into Anionic Tumor‐Mimetic Membranes

**FIGURE 5:**
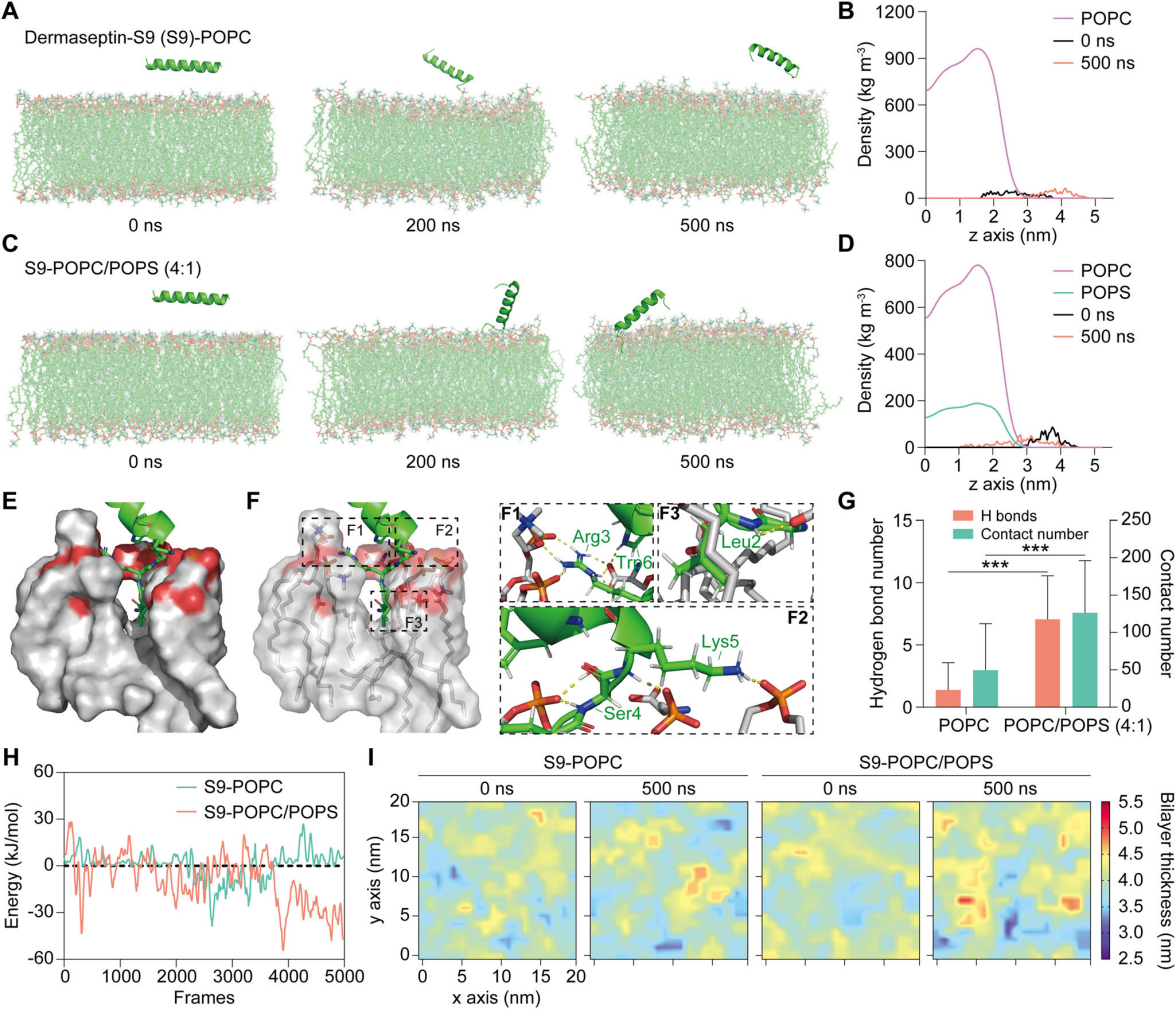
MD simulations of S9 interactions with model membranes. (A) MD simulation of S9 interacting with a zwitterionic POPC membrane over 500 ns. (B) Longitudinal distribution density of S9 at 0 and 500 ns on the POPC membrane. (C) MD simulation of S9 interacting with a negatively‐charged POPC/POPS (4:1) membrane over 500 ns. (D) Longitudinal distribution density of S9 at 0 and 500 ns on the POPC/POPS (4:1) membrane. (E) Representative snapshot of the interaction between S9 and the POPC/POPS (4:1) membrane, shown in both cartoon and stick representations. The peptide is shown in green, with interacting lipid residues highlighted in red. (F) Detailed view of hydrogen bonding between S9 and the POPC/POPS (4:1) membrane at 500 ns, with yellow dashed lines indicating hydrogen bonds. (G) Quantification of the average number of hydrogen bonds and atomic contacts between S9 and both membrane types over the 500‐ns simulation. (H) Calculated interaction energy between S9 and each membrane system during the 500‐ns simulation (Each frame was recorded at an interval of 100 ps). (I) 2D heatmap showing the average membrane thickness at 500 ns after S9 treatment, illustrating peptide‐induced membrane perturbation. Data are presented as mean ± SD, ***P < 0.001.

We performed molecular dynamics (MD) simulations to examine S9 interactions with zwitterionic mammalian‐mimetic and anionic tumor‐mimetic bilayers. During the 500 ns simulation, S9 showed no stable contact with the zwitterionic membrane over the entire trajectory (Figure 5A). In contrast, S9 contacted the anionic membrane by 200 ns and penetrated more deeply thereafter (Figure 5C). Mass‐density profiles and peptide‐bilayer center‐of‐mass distances indicated partial embedding in the anionic bilayer, while S9 remained distant from the zwitterionic bilayer at 500 ns (Figure 5B,D). At 500 ns, S9 formed 12 hydrogen bonds (dotted yellow lines) with the negatively‐charged membrane, whereas no peptide‐membrane interaction was observed for the zwitterionic system (Figure 5E, F; Figure S3). Across the full simulation, both the average number of hydrogen bonds and the number of peptide‐lipid atomic contacts were higher for the tumor‐mimetic membrane (Figure 5G). Interaction‐energy analysis also indicated a progressive increase in attractive peptide‐membrane interactions in the anionic system, with no consistent trend in the zwitterionic system (Figure 5H). A heatmap of membrane thickness further revealed local thinning and lipid redistribution in the anionic bilayer, consistent with peptide‐induced perturbation (Figure 5I). CD measurements further showed that the secondary structure of S9 was influenced by the membrane environment. In the presence of 1‐palmitoyl‐2‐oleoyl‐sn‐glycero‐3‐phosphocholine (POPC)/1‐palmitoyl‐2‐oleoyl‐sn‐glycero‐3‐phospho‐L‐serine (POPS) (4:1), S9 exhibited a more pronounced α‐helical signature, whereas the spectral change was less evident in the POPC system (Figure S4), consistent with its preferential interaction with anionic tumor‐mimetic membranes. Together, these simulations indicate that S9 selectively interacts with anionic tumor‐mimetic membranes and induces local bilayer perturbation. The preferential insertion of S9 into the anionic membrane, together with the increased hydrogen bonding, atomic contacts, and membrane thinning, supports the interpretation that S9 acts through a membrane‐disruptive mode of action. Although the current simulations do not define a specific pore architecture or oligomerization state, they suggest that S9 perturbs tumor‐mimetic membranes through a dynamic process involving transient pore‐like defects or local membrane destabilization [52, 53, 54].

### 2.7 S9 Induces Membranolysis and ICD

**FIGURE 6:**
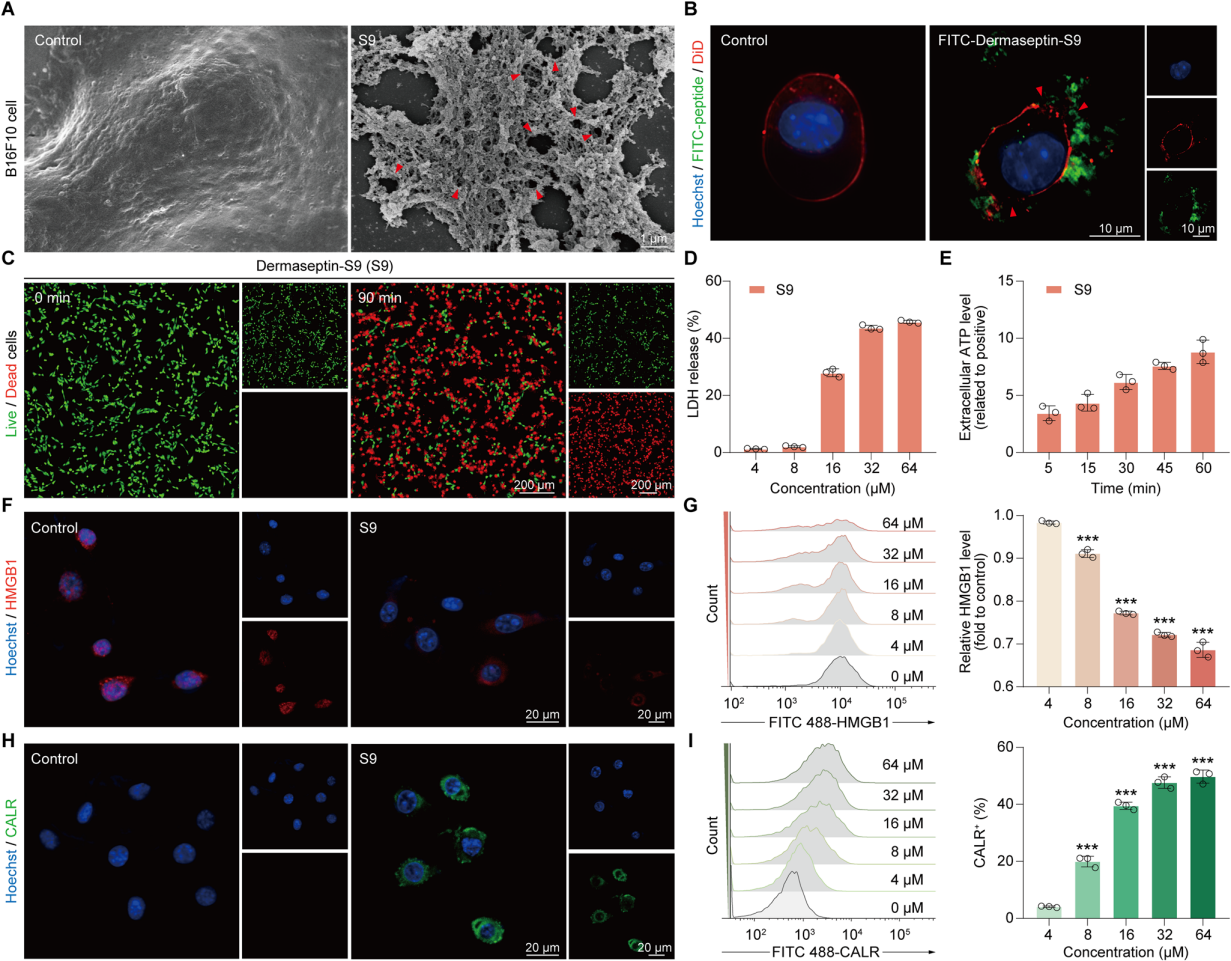
S9‐mediated oncolysis and ICD in B16F10 cells. (A) Representative SEM images exhibiting the lytic effect of S9 (32 µM for 4 h) on B16F10 cells. (B) SRFM images showing membrane disruption in B16F10 cells following 32 µM S9 treatment for 4 h. (C) Live/dead staining of B16F10 cells after exposure to S9 (32 µM) for 90 min. (D) LDH release from B16F10 cells treated with various concentrations of S9 for 24 h. (E) ATP secretion levels measured at multiple time points following S9 (64 µM) treatment. (F) Representative confocal immunofluorescence images showing HMGB1 release in B16F10 cells treated with 32 µM S9 for 6 h. (G) FCM analysis and quantification of HMGB1 release in response to S9 treatment (4‐64 µM) for 6 h. (H) Representative confocal immunofluorescence images showing CALR surface exposure in B16F10 cells treated with 32 µM S9 for 6 h. (I) FCM analysis and quantification of CALR exposure in B16F10 cells following S9 treatment (4‐64 µM) for 6 h. Data are presented as mean ± SD (n = 3 independent experiments). Statistical significance: ***P < 0.001 versus Control.

We next evaluated whether S9 elicits oncolysis and hallmarks of ICD. OLPs disrupt tumor plasma membranes and promote the release or exposure of damage‐associated signals, including adenosine triphosphate (ATP), high‐mobility group box 1 (HMGB1), and calreticulin (CALR), thereby activating antitumor immunity. To assess membranolysis, scanning electron microscopy (SEM) revealed S9‐treated B16F10 cells exhibited irregular cell morphology and fragmented debris (Figure 6A). Subsequently, we labeled S9 with FITC through a flexible 6‐aminohexanoic acid linker to minimize potential steric interference with peptide‐membrane interactions (Figure S19A, B). Cell viability assays showed that fluorescein isothiocyanate (FITC)‐labeled S9 retained substantial antiproliferative activity in B16F10 cells (Figure S19C). Consistently, super‐resolution fluorescence microscopy (SRFM) showed that FITC‐labeled S9, together with DiD‐stained membranes, visualized overt membrane disruption in S9‐treated B16F10 cells, whereas control cells retained intact membrane morphology (Figure 6B). Live‐dead imaging showed that S9 caused a progressive loss of calcein‐AM fluorescence with a concomitant rise in propidium iodide (PI) uptake, culminating in widespread cell death by 90 min (Figure 6C). Consistently, S9 induced dose‐dependent release of lactate dehydrogenase (LDH), a cytosolic enzyme liberated upon membrane damage (Figure 6D).

We then examined ICD markers. S9 triggered time‐dependent ATP release from B16F10 cells (Figure 6E). Immunofluorescence showed nuclear HMGB1 in control cells but prominent extracellular HMGB1 after S9 exposure (Figure 6F), which was corroborated by flow cytometry (FCM) demonstrating a concentration‐dependent decrease in intracellular HMGB1 (Figure 6G). Moreover, S9 promoted CALR exposure on the tumor cell surface, as evidenced by immunofluorescence (Figure 6H) and quantitative FCM (Figure 6I). Together, these results establish S9 as a potent OLP that rapidly disrupts tumor membrane integrity and induces ICD signals, providing a mechanistic basis for downstream immune activation.

### 2.8 Antitumor Efficacy of S9 In Vivo

**FIGURE 7:**
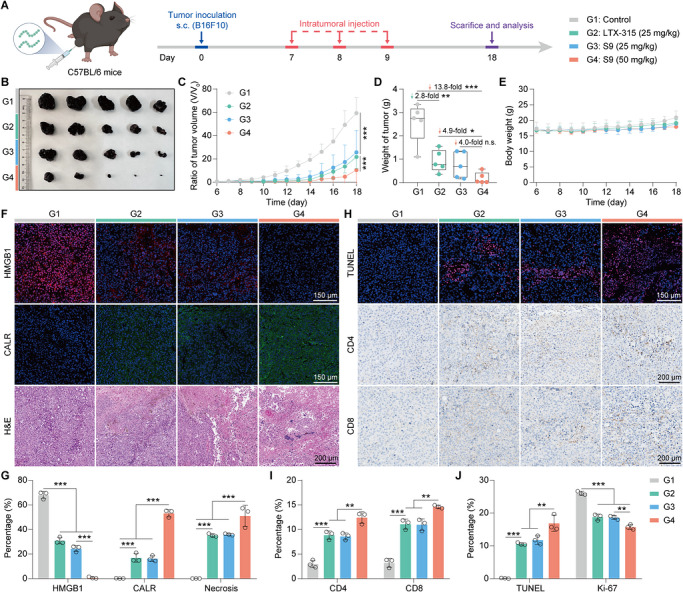
In vivo antitumor efficacy and immunomodulatory effects of S9 in a B16F10 tumor model. (A) Schematic illustration of tumor establishment and treatment schedule in the B16F10 model. (B) Images of excised tumors from each treatment group (n = 5). (C) Tumor growth curves showing relative volume changes across groups (n = 5). (D) Tumor weights following treatment (n = 5). (E) Body weight of mice monitored throughout the treatment period (n = 5). (F, G) Representative images and quantitative analysis of immunofluorescence staining for CALR and HMGB1, and hematoxylin and eosin (H&E) staining of tumor sections. (H–J) Representative images and quantification of terminal deoxynucleotidyl transferase dUTP nick‐end labeling (TUNEL) staining, and immunohistochemical staining for CD4^+^ and CD8^+^ T cells in tumor tissues. Data are presented as mean ± SD, *P < 0.05, **P < 0.01, and ***P < 0.001.

Given the potent in vitro oncolytic activity of S9, we evaluated its efficacy in a B16F10 melanoma model. When tumors reached 30–50 mm^3^, mice were randomized into four groups (phosphate‐buffered saline (PBS), LTX‐315, low‐dose S9, high‐dose S9; n = 5 per group) and treated three times as scheduled in Figure 7A. Body weight remained stable across groups, and routine blood indices showed no significant differences, indicating a favorable safety profile for S9 (Figure 7E; Figure S5). Notably, S9 suppressed melanoma growth in a dose‐dependent manner. At an equivalent dose, S9 achieved tumor control comparable to LTX‐315 (Figure 7B). High‐dose S9 significantly reduced tumor volume and final tumor weight versus PBS, with a tumor growth inhibition of 92.72%, demonstrating robust in vivo antitumor activity (Figure 7C,D). To assess in vivo ICD, tumor sections were stained for CALR and HMGB1. High‐dose S9 increased both markers, consistent with ICD induction observed in vitro (Figure 7F,G). Hematoxylin‐eosin staining revealed extensive necrosis in OLP‐treated tumors compared with controls, while immunohistochemical analyses showed increased apoptosis and reduced proliferation (Figure 7J; Figure S6). Moreover, S9 enhanced intratumoral T‐cell infiltration, with significant increases in CD4^+^ and CD8^+^ subsets compared with other groups (Figure 7H,I). Together, these data indicate that S9 is well tolerated in vivo and exerts potent antitumor effects coupled to immunogenic remodeling of the tumor microenvironment.

## 3 Discussion

OLPs are emerging as compelling alternatives to conventional cancer therapies, capable of directly lysing tumor cells and stimulating antitumor immunity even in resistant settings. However, their discovery still relies heavily on labor‐intensive extraction, synthesis, and phenotypic screening, which constrain throughput and escalate costs. ML offers a powerful means to navigate the complex peptide sequence–structure–function landscape and accelerate the discovery of mechanism‐aligned OLPs. Continued progress will require addressing data sparsity, minimizing false positives, and improving translational readiness so that diverse, safe, and effective OLPs can advance to the clinic.

Most existing ACP or AMP predictors are trained on broad activity labels using a single classifier, while many generative models expand sequence space without guaranteeing mechanistic fidelity. In this study, we introduce MISPOP, a mechanism‐guided pipeline that integrates three complementary learners and advances a sequence only when all models concur, thereby reducing false positives when experimental validation is costly. MISPOP further incorporates key biophysical priors central to oncolysis—amphiphilicity, hydrophobicity, net charge, and helical bias. The use of SHAP interpretation links predictions to feature‐level contributions, yielding smaller shortlists enriched for peptides with membrane‐disruptive potential. On a held‐out test set, the high‐confidence ensemble reduced the false‐positive rate to 1.22%, compared with 7.41% for majority voting. Applied prospectively to a natural peptide library of 1033 sequences with no prior antitumor annotation, MISPOP identified 16 candidates, of which five peptides with high predicted helicity were synthesized for experimental validation.

Wet‐lab assays confirmed antiproliferative activity and acceptable hemolytic profiles among the shortlisted peptides, with S9 displaying the most favorable therapeutic index. MD simulations placed S9 within anionic, tumor‐mimetic bilayers while sparing zwitterionic membranes, accompanied by stable electrostatic and hydrogen‐bond contacts and local membrane thinning‐signatures of selective membranolysis. Cellular studies corroborated these findings, showing rapid membrane disruption and induction of the ICD triad comprising ATP release, HMGB1 translocation, and surface CALR exposure. In vivo, these same ICD hallmarks were observed within tumors, alongside robust growth inhibition and elevated CD4^+^/CD8^+^ T cell infiltration, without systemic toxicity. The consistency across simulation, cellular, and animal levels establishes a direct mechanistic link between the biophysical priors embedded in MISPOP and realized pharmacological efficacy. This coherence not only validates the computational strategy but also informs future optimization—highlighting the need to maintain cationic charge and amphiphilic patterning sufficient for anionic membrane insertion while minimizing hemolysis.

Despite these advances, a key limitation lies in molecular representation. The current models do not yet encode post‐translational modifications, D‐amino acids, stapled or cyclic scaffolds, peptoids, or β‐peptides. The representation captures secondary‐structure tendencies but not 3D conformations or self‐assembly behaviors, both of which influence membrane insertion, selectivity, and hemolytic potential. Future iterations will expand the token set to include modified monomers and integrate structural and aggregation descriptors derived from MD and biophysical assays. MISPOP's modular design allows flexible integration of alternative priors and labels, enabling its adaptation to other mechanism‐defined peptide classes. Moreover, it can serve as a mechanism‐aware filter for generative models or operate within an active‐learning loop that jointly optimizes potency, hemolysis, and stability.

Collectively, MISPOP establishes a reproducible, mechanism‐aligned route from sequence to efficacy, bridging computational prediction with experimental validation. This framework provides a practical foundation for the rational discovery and optimization of next‐generation peptide immunotherapeutics.

## 4 Experimental Section

### 4.1 Data Collection and Preprocessing

To construct datasets for OLP model development, we curated experimentally validated ACP sequences from publicly available ACP‐ and AMP‐related repositories, including DBAASP [55], DRAMP [56], SATPdb [57], APD3 [58], CAMP [59], CancerPPD [60], and EROP‐Moscow [61]. We also included published ACP benchmark datasets from ACPred [62], CpACpP [63], ACP‐DL [64], AntiCP2.0 [36], and MLACP [42] to improve the coverage of experimentally validated ACP sequences. These ACPs were used as the positive dataset. For the negative dataset, we retrieved reviewed UniProt sequences with lengths of 4–30 amino acids [65]. To minimize contamination by potential ACPs or AMPs, we excluded sequences annotated with keywords related to anticancer, antitumor, apoptosis, programmed cell death, antimicrobial, antibiotic, toxic, defensin, antiviral, antifungal, antibacterial, secreted peptides/proteins, and other defense‐related terms. Only sequences composed of natural amino acids were retained, and sequences containing B, J, O, U, X, or Z were removed. To reduce homology bias and avoid overfitting, both the positive and negative datasets were filtered using CD‐HIT [66] at a 90% sequence identity threshold. This process yielded 1921 experimentally validated ACPs and 1253 negative sequences that were not annotated as anticancer or antimicrobial peptides. For transfer learning model construction, AMP sequences were collected using a similar strategy. Data were obtained from AMP‐related databases, including CAMP, LAMP2 [67], DRAMP, DBAASP, and CancerPPD, as well as published AMP datasets, including AMPfun [68], AmPEP [69], and AniAMPpred [70]. After removal of redundant and noncanonical sequences, 9206 unique AMP sequences were retained for further analysis. The ACP dataset was randomly split into training and test sets at a ratio of 9:1, yielding 1729 ACPs and 1128 non‐ACPs in the training set and 192 ACPs and 125 non‐ACPs in the test set. Overlapping peptides between the ACP dataset and the AMP dataset used for transfer learning were deliberately retained to allow the TL model to learn shared sequence features, but this did not affect the independence of the ACP training and test sets.

### 4.2 Feature Extraction and Dimensionality Reduction

In the process of ACP modeling, 8 sequence‐based features were utilized, including binary profile features (BPF) [71], 21‐bit features (TBF) [72], amino acid composition (AAC) [73], atomic composition (ATC) [74], reduced amino acid composition (RAAC) [75], physicochemical property composition (PPC), BLOSUM62 [34], and physicochemical properties (DBAASP‐PP). Detailed descriptions of the feature extraction methods are provided in the Supporting Information. All the sequences in the training set were first encoded into a feature matrix of N×1913 (N represents the number of sequences) through feature embedding methods. Then, the feature matrix was normalized utilizing the min–max normalization approach. To minimize redundancy and prevent overfitting, the mRMR was applied to rank features by classification relevance [76]. The top 800 ranked features were retained. Subsequently, the random forest algorithm was used to refine feature selection based on the MCC. Through fivefold cross‐validation, the optimal subset of 405 features was identified, which yielded the highest MCC and was ultimately used to construct the embedding space. Specifically, the mRMR method was also applied to the AMP dataset to extract the optimal features, and these AMP‐specific features were incorporated into the training process of the final TL model.

### 4.3 Construction, Optimization, and Evaluation of Computational Models

Various ML algorithms were employed in this study to construct predictive models for identifying potential OLPs. XGBoost, a widely used ensemble learning algorithm, was employed due to its robust performance and ability to effectively manage diverse data types [32]. DNN is a deep learning architecture inspired by biological nervous networks that simulates the processing mechanisms of the neural system of the human brain to handle complex information [31]. The DNN models were built using the PyTorch framework, a Python‐based deep learning library. In addition, TL, a prominent paradigm in ML, was applied to leverage knowledge from a related source task to enhance learning in the target task. Instead of training a model from scratch on the target task with limited data, TL allows the reuse of pretrained model representations derived from related datasets. Detailed procedures for hyperparameter optimization during model construction are presented in the Supporting Information.

Model performance was systematically evaluated across six standard metrics: accuracy (Acc), precision (Pre), sensitivity (Sen), specificity (Spe), MCC, and area under the ROC curve (AUC). These evaluation metrics are based on the following equations:

$$\mathrm{Acc}\;=\frac{\mathrm{TP}+\mathrm{TN}}{\mathrm{TP}+\mathrm{FP}+\mathrm{TN}+\mathrm{FN}}$$

$$\mathrm{Pre}\;=\frac{\mathrm{TP}}{\mathrm{TP}+\mathrm{FP}}$$

$$\mathrm{Sen}\;=\frac{\mathrm{TP}}{\mathrm{TP}+\mathrm{FN}}$$

$$\mathrm{Spe}\;=\frac{\mathrm{TN}}{\mathrm{TN}+\mathrm{FP}}$$

$$\mathrm{MCC}\;=\frac{\mathrm{TP}\times \mathrm{TN}-\mathrm{FP}\times \mathrm{FN}}{\sqrt{\left(\mathrm{TP}+\mathrm{FP}\right)\left(\mathrm{TP}+\mathrm{FN}\right)\left(\mathrm{TN}+\mathrm{FP}\right)\left(\mathrm{TN}+\mathrm{FN}\right)}}$$

TP (True Positive) refers to the number of positive samples correctly predicted as positive, while TN (True Negative) indicates the number of negative samples correctly predicted as negative. FP (False Positive) represents the number of negative samples incorrectly predicted as positive, while FN (False Negative) represents the number of positive samples incorrectly predicted as negative.

### 4.4 Experimental Materials and Reagents

All chemical reagents were purchased from Greagent and Adamas‐beta and were used exclusively for research purposes. Rink Amide resin (0.34 mmol/g loading) was purchased from Tianjin Nankai Hecheng Science & Technology Co., Ltd. The medium for cell culture media were supplied by MeilunBio and Yeasen Biotechnology (Shanghai) Co., Ltd.

### 4.5 Cell Culture and Animal Models

The melanoma cell lines B16F10 and A375, as well as the breast tumor cell line 4T1, were purchased from the Cell Bank of the Chinese Academy of Sciences (Shanghai, China). Cells were cultured in Dulbecco's modified Eagle's medium with 10% (v/v) fetal bovine serum and antibiotics (100 µg/mL streptomycin and 100 units/mL penicillin). The animal experiments used female C57 mice (age: 3–4 weeks, weight: 18–22 g) sourced from the Shanghai Model Organisms Center, Inc. The arrangements for the animal study were approved by the ethical committee of the Shanghai Model Organisms Center, Inc (IACUC No: 2023‐0015‐1).

### 4.6 Peptide Synthesis

All peptides were synthesized using the standard 9‐fluorenylmethoxycarbonyl (Fmoc)‐based solid‐phase peptide synthesis (SPPS) method, following previously established protocols [19]. Briefly, Rink Amide resin was first swollen in dichloromethane (DCM), and the Fmoc group was removed using a solution of 20% piperidine/N,N‐Dimethylformamide (DMF)/0.1 mol/L Oxyma pure. Amino acid coupling was carried out with Fmoc‐AA‐OH (5 equiv.), N, N’‐diisopropylcarbodiimide (DIC, 5 equiv.), and Oxyma pure (5 equiv.) dissolved in N‐Methyl‐2‐pyrrolidone (NMP) solvent at 60°C for 20 min. After each coupling, the remaining reaction solution was washed with DCM and DMF. The deprotection–coupling–washing cycle was repeated until full peptide assembly was completed. Following final Fmoc deprotection, a cocktail reagent (trifluoroacetic acid, triisopropylsilane, and H_2_O at a 95:2.5:2.5 ratio) was added into the resin to cleave the peptide at room temperature for 2 h. The resin was filtered, and crude peptides were precipitated with cold diethyl ether. After centrifugation at 4000 rpm for 5 min, the supernatant solution was discarded, and the crude peptides were dried under N_2_. Finally, the crude peptides were purified and analyzed via high‐performance liquid chromatography (HPLC) and mass spectrometry (MS).

### 4.7 Cell Viability Assay

The antitumor activity of peptides was evaluated using the Cell Counting Kit‐8 (CCK‐8) assay. Tumor cells were seeded in 96‐well plates at a density of 5 × 10^3^ cells/well and cultured for 24 h. The medium was then replaced with fresh medium containing peptides with various concentrations, while control wells received medium only. After 24 h, fresh serum‐free medium containing 10% CCK‐8 solution was added to each well. Absorbance was measured at 450 nm using a microplate reader (BioTek Cytation 5, USA).

### 4.8 Red Blood Cell Hemolysis Assay

The hemolytic side effect of peptides was assessed by quantifying hemoglobin release from freshly isolated mouse erythrocytes. Whole blood was diluted in normal saline (NS), and erythrocytes were collected by centrifugation and washed with NS. Erythrocytes were resuspended to 4% (v/v) in NS and plated into 96‐well plates. Peptides were added at final concentrations of 1, 2, 4, 8, 16, 32, 64, 128, and 256 µM, and 0.1% Triton X‐100 served as a positive control. After incubation at 37°C for 1 h, plates were centrifuged (1000 g, 10 min, 4°C) and the supernatant transferred to new plates. The hemolysis of peptides was determined by measuring absorbance at 570 nm and calculated using the formula: (Apeptide − ANS)/(Apositive − ANS) × 100%.

### 4.9 Preparation of Model Membranes

Model membranes composed of POPC and POPC:POPS (4:1) were prepared by the thin‐film dispersion strategy to simulate tumor and normal mammalian cells, respectively. Briefly, lipid materials were completely dissolved in chloroform, transferred them to a round‐bottom flask and obtained phospholipid film using a vacuum evaporation method. Purified water was used to hydrate the lipid film, and then homogeneous model membranes were obtained via a polycarbonate filter (with a 200 nm pore size).

### 4.10 CD Spectroscopy Analysis

Peptides were dissolved in purified water to 100 µM and loaded into a 1 mm path‐length quartz cuvette. For the determination of the secondary structure of peptides cultured with model membrane environment, the ratio between the peptides and model membranes were set at 1/32, with purified water serving as the solvent background. CD spectra were recorded at room temperature using a BRIGHT TIME Chirascan spectropolarimeter (Applied Photophysics, UK) over 190–260 nm with a 0.5 nm step and 20 nm/min scan rate. Raw spectra were corrected by subtracting the buffer baseline and converted to a uniform scale of molar ellipticity. Each spectrum was smoothed five times using the instrument's standard parameters without significant distortion.

### 4.11 MD Simulation

In this work, all peptides' 3D structures were modelled using the PEPFOLD4 webservice, and the bilayer membrane model was constructed in the CHARMM‐GUI webservice. For the simulations of tumor and mammalian cells, the compositions were POPC:POPS (4:1) and POPC, respectively. Each peptide was positioned 3 nm above the membrane surface, and the system was solvated in a water box of 10 nm by 10 nm by 15 nm with 0.1 M concentration of NaCl. MD simulations were performed with a 2 fs integration step, constraining hydrogen bonds via the LINCS algorithm. Electrostatics were performed with the particle‐mesh Ewald method, and non‐bonded interactions truncated at 10 Å with neighbor lists updated every 10 steps. Pressure coupling was maintained at 1 bar via the Parrinello–Rahman barostat, and temperature at 303.15 K with the V‐rescale thermostat. Following energy minimization and equilibration, 500 ns production runs were conducted. Trajectories were visualized with PyMOL, and the lipid‐peptide interactions during the simulation were analyzed.

### 4.12 Live/Dead Cell Staining

Tumor cell viability was assessed using a Viability/Cytotoxicity Assay Kit (Beyotime, C2015S). B16F10 cells were seeded into a 96‐well plate at a density of 1 × 10^4^ cells/well and incubated for 24 h. Cells were stained with Calcien‐AM (Ex: 488 nm and Em: 515 nm) and then treated witht serum‐free medium containing S9 (32 µM) and PI (Ex: 535 nm and Em: 617 nm). Live/dead fluorescence images were captured at 0, 60, and 90 min using a SparkCyto cytometer.

### 4.13 LDH Release Assay

Cytotoxicity was further evaluated by quantifying lactate dehydrogenase (LDH) release with the LDH Release Assay Kit‐WST (DOJINDO, CK‐12). B16F10 cells were seeded into a 96‐well plate at a density of 5 × 10^3^ cells/well. After 24 h of incubation, the cells were treated with a medium containing S9 for 24 h. Serum‐free medium served as the negative control, whereas cells with lysis buffer were used as the positive control. The working solution was added and incubated for 30 min, and absorbance was measured at 490 nm using Cytation 5.

### 4.14 Observation of the Oncolytic Process by Fluorescence Microscope

B16F10 cells were seeded in a glass‐bottom dish at a density of 5 × 10^4^ cells per well and incubated for 24 h. Afterward, the cells were treated with FITC‐labeled S9 (FITC‐S9) for 4 h, followed by staining with DiD and Hoechst 33342 to visualize cell membranes and nuclei, respectively. The lytic activity of FITC‐S9 on tumor cells was observed using the GEDeltaVision OMX SR.

### 4.15 Observation of the Oncolytic Effect by SEM

B16F10 cells were seeded on sterile coverslips at a density of 1.5 × 10^5^ cells and incubated for 24 h. Subsequently, S9 (concentration of twofold IC_50_) was added into the well and serum‐free medium as the control. After 4 h of treatment, the cells were washed with PBS and then fixed with the 2.5% glutaraldehyde solution. Fixed cells were gradient dehydrated with ethanol (30%, 50%, 70%, 90%, and 100%), and the samples were dried and observed by SEM (Hitachi S‐4800, Japan).

### 4.16 ATP Release Assay

ATP release was quantified using the Enhanced ATP Assay Kit (S0027). B16F10 cells were seeded into 96‐well plates at a density of 1 × 10^4^ cells/well and incubated overnight. Cells were treated with S9 for varying time intervals (5, 15, 30, 45, and 60 min). Untreated cells served as the negative control, while cells with the lysis buffer‐treated cells served as the positive control. The supernatants were mixed with working solution, and analyzed on a Tecan Spark luminometer (Tecan Spark, Switzerland).

### 4.17 Immunofluorescence Staining of CALR

B16F10 cells were seeded in a glass‐bottom dish at a density of 1 × 10^5^ cells per well. After 24 h of incubation, the cells were treated with S9 for an additional 6 h, whereas the control cells received medium only. After treatment, cells were washed with cold PBS, fixed in 4% paraformaldehyde for 20 min, and blocked nonspecific binding with 3% bovine serum albumin (BSA) solution for 1 h. After washing with PBS, Cells were stained with CALR (D3E6) XP Rabbit mAb (CST, 12238) for 1 h, followed by Hoechst 33342 nuclear staining. Surface detection of CALR was observed using the GE DeltaVision OMX SR. FCM was also used to detect CALR exposure of S9‐treated tumor cells. B16F10 cells were seeded into 12‐well plates at a density of 100 000 cells/well. After 24 h of incubation, the cells were treated with different concentrations of S9 for 6 h. Subsequently, cells were digested and collected, blocked nonspecific binding sites, and stained with CALR antibody. The level of CALR exposure of tumor cells was quantified by FCM.

### 4.18 Immunofluorescence Staining of HMGB1

For HMGB1 detection, B16F10 cells were seeded in a glass‐bottom dish at a density of 1 × 10^5^ cells per well. After 24 h of incubation, the cells were treated with S9 for 6 h. Cells were washed, fixed in 4% paraformaldehyde for 20 min, permeabilized with 1% Triton X‐100 for 5 min, and blocked with 1% BSA for 1 h. Afterward, cells were incubated with an anti‐HMGB1 antibody (Abcam, ab227168) at 4°C for 16 h, followed by incubation with an Alexa Fluor 594‐conjugated secondary antibody (Yeasen, 33112) for 2 h at room temperature. Hoechst 33342 was used for nuclear counterstaining, and images were acquired using the GE DeltaVision OMX SR. HMGB1 release was also quantified by FCM under similar conditions, except that permeabilization and fixation were omitted after blocking.

### 4.19 In Vivo Antitumor Therapy

The female C57 mice were subcutaneously inoculated with 5 × 10^5^ B16F10 cells to establish a melanoma model. Once the average tumor volume reached about 30–50 mm^3^, mice were randomly divided into 4 groups (n = 5) and received intratumoral injections of PBS, LTX‐315 (25 mg/kg), low‐dose S9 (25 mg/kg), or high‐dose S9 (50 mg/kg) for 3 consecutive days. The tumor volume was calculated daily using the formula (length × width2)/2, along with body weight monitoring. At the end of the experiment, mice were euthanized, and their tumors were collected and fixed with 4% paraformaldehyde. Routine safety indices were assessed in the blood, and tumor slices were stained with H&E, HMGB1, CALR, TUNEL, CD4, CD8, and Ki‐67.

### 4.20 Statistical Analysis

All data were analyzed using GraphPad Prism 10.1.2 software. Differences between groups were evaluated using Student's t‐test or two‐way ANOVA analysis method. Quantitative data were considered significantly different when the values of *P < 0.05, **P < 0.01, and ***P < 0.001.

## Conflicts of Interest

The authors declare no conflicts of interest.

## Supporting information




**Supporting File**: advs75652‐sup‐0001‐SuppMat.docx.

## Data Availability

The data that supports the findings of this study are available in the supplementary material of this article.
