# Supplementary material for: Mechanism‐Informed Machine Learning Enables Discovery of Oncolytic Peptides for Cancer Immunotherapy
Source: Adv Sci (Weinh). 2026 May 12:e75652. Online ahead of print. doi: 10.1002/advs.75652 (PMC13336043; doi:10.1002/advs.75652)
Supplement: Supplementary file 1 — Supporting File: advs75652‐sup‐0001‐SuppMat.docx. [file ADVS-9999-e75652-s001.docx]

Supporting Information

Mechanism-Informed Machine Learning Enables Discovery of Oncolytic Peptides for Cancer Immunotherapy

Wen Zhang^1^, Shengxin Lu^1^, Guangyong Zheng^1^, Shensuo Li^1^, Hongyu Chen, Mei Hong,

Xiangru Zhou, Ruotian Tang, Ye Wu*, Weidong Zhang*, Dong Lu*, Xin Luan*

W. Zhang, S.-X. Lu, G.-Y. Zheng, H.-Y. Chen, M. Hong, X.-R. Zhou, R.-T. Tang,

Y. Wu, D. Lu, X. Luan

State Key Laboratory of Discovery and Utilization of Functional Components in Traditional Chinese Medicine, Shanghai Frontiers Science Center of Chinese Medicine Chemical Biology, Institute of Interdisciplinary Integrative Medicine Research and Shuguang Hospital, Shanghai University of Traditional Chinese Medicine, Shanghai 201203, China.
E-mail: wuye@shutcm.edu.cn; wdzhangy@hotmail.com; ludong@shutcm.edu.cn; luanxin@shutcm.edu.cn

W.-D. Zhang

Shanghai Institute of Infectious Diseases and Biosafety, Institute of Interdisciplinary Integrative Medicine Research, Shanghai University of Traditional Chinese Medicine, Shanghai 201203, China.

W.-D. Zhang

School of Pharmacy, Naval Medical University, Shanghai 200433, China.

S.-S. Li

West China School of Public Health and West China Fourth Hospital, State Key Laboratory of Biotherapy, Sichuan University, Chengdu 610041, China.

W.-D. Zhang

State Key Laboratory for Quality Ensurance and Sustainable Use of Dao-di Herbs, Institute of Medicinal Plant Development, Chinese Academy of Medical Sciences and Peking Union Medical College, Beijing 100700, China.

^1^ W. Zhang, S.-X. Lu, G.-Y. Zheng, and S.-S. Li contributed equally to this work.

# 1. Experimental methods

## 1.1 Binary profile features (BPF)

The 20 natural amino acids are represented using the standard single-letter code (A, C, D, E, F, G, H, I, K, L, M, N, P, Q, R, S, T, V, W and Y), and each amino acid is encoded with a tuple with 20 binary elements that comprise 1 and 0. For instance, alanine (A) is encoded as (1, 0, 0, 0, 0, 0, 0, 0, 0, 0, 0, 0, 0, 0, 0, 0, 0, 0, 0, 0), cysteine (C) as (0, 1, 0, 0, 0, 0, 0, 0, 0, 0, 0, 0, 0, 0, 0, 0, 0, 0, 0, 0), and aspartic acid (D) as (0, 0, 1, 0, 0, 0, 0, 0, 0, 0, 0, 0, 0, 0, 0, 0, 0, 0, 0, 0), etc. Since the longest anticancer peptides (ACPs) in the dataset consist of 30 amino acids, each peptide is converted into a 600-dimensional vector. Peptides shorter than 30 residues are padded with zeros to maintain consistent dimensionality.

## 1.2 Twenty-one-bit features

The standard amino acid alphabet is qualitatively categorized into seven classes based on physicochemical properties: charge, hydrophobicity, normalized Van der Waals volume, polarity, polarizability, secondary structures, and solvent accessibility(1). For each property, amino acids were divided into three distinct groups (Table S1), resulting in a total of 21 groups. Following a binary profile framework similar to BPF encoding, each amino acid was encoded as a 21-dimensional binary vector, where the presence of the amino acid in a specific group is indicated by 1 and absence by 0. Padding is applied to peptide sequences shorter than 30 amino acids to ensure consistent feature dimensionality. Therefore, the feature vector for a peptide is 630-dimensional.

**Table S1.**

Groups of amino acids based on the physicochemical properties of the 21-bit features.

| **Physicochemical Properties** | **Group 1** | **Group 2** | **Group 3** |
| --- | --- | --- | --- |
| Charge | A, C, F, G, H, I, L, M, N, P, Q, S, T, V, W, Y  (Neutral) | D, E  (Negatively charged) | K, R  (Positively charged) |
| Hydrophobicity | C, F, I, L, M, V, W  (Hydrophobicity) | A, G, H, P, S, T, Y  (Neutral) | D, E, K, N, Q, R  (Polar) |
| Normalized van Der Waals Volume | A, C, D, G, P, S, T  (0-2.78) | E, I, L, N, Q, V  (2.95-4.0) | F, H, K, M, R, W, Y  (4.03-8.08) |
| Polarity | C, F, I, L, M, V, W, Y  (4.9-6.2) | A, G, P, S, T  (8.0-9.2) | D, E, H, K, N, Q, R  (10.4-13.0) |
| Polarizability | A, D, G, S, T  (0-0.108) | C, E, I, L, N, P, Q, V  (0.128-0.186) | F, H, K, M, R, W, Y  (0.219-0.409) |
| Secondary Structures | D, G, N, P, S  (Coil) | A, E, H, K, L, M, Q, R  (Helix) | C, F, I, T, V, W, Y  (Strand) |
| Solvent Accessibility | A, C, F, G, I, L, V, W  (Buried) | H, M, P, S, T, Y  (Intermediate) | D, E, K, N, R, Q  (Exposed) |

## 1.3 Amino acid composition (AAC) and atomic composition (ATC)

AAC is defined as the frequency of each amino acid in a peptide sequence divided by the total sequence length. ATC is another encoding method that calculates the frequency of each type of atom (C, H, N, O, S) present in natural amino acids(2), resulting in a 5-dimensional feature vector.

## 1.4 Reduced amino acid composition (RAAC)

Hydrophilic and hydrophobic residues have a significant impact on the overall structure of peptides*(3)*. Accordingly, the 20 standard amino acids were classified into six groups based on hydrophilicity **(Table S2)***(4, 5)*. Each peptide sequence can be represented by a string of 6 characters corresponding to these groups, from which the dipeptide composition of the six-character representation can be derived. The representation is as follows:

$$F=[f_{1},f_{i}, \cdots\text{ },f_{36}]^{T}$$

where $f_{i}(i=1, 2\cdots36)$ is the absolute occurrence frequency of the 36 hydropathy dipeptides, calculated as follows:

$$f_{i}=\frac{n_{i}}{L-1}$$

where $n_{i}$ is the occurred count of the 36 hydropathy dipeptides of a peptide sequence and L is the sequence length.

**Table S2**.

Classification of amino acids based on reduced amino acid composition features.

| **Classification** | **Abbreviation** | **Amino acids** |
| --- | --- | --- |
| Strongly Hydrophilic or Polar | L | R, D, E, N, Q, K, H |
| Strongly Hydrophobic | B | L, I, V, A, M, F |
| Weakly Hydrophilic or Weakly Hydrophobic | W | S, T, Y, W |
| Proline | P | P |
| Glycine | G | G |
| Cysteine | C | C |

## 1.5 Physicochemical property composition (PPC)

The 20 natural amino acids were grouped into 10 distinct categories (Table S3)(6), and the composition percentages of each group was calculated, allowing each peptide sequence to be converted into a 10-dimensional vector.

**Table S3**.

Ten amino acid groups according to physicochemical property composition.

| **Groups** | **Amino acids** |
| --- | --- |
| Hydrophobic Amino Acids | C, V, L, I, M, F, W |
| Aliphatic Amino Acids | I, L, V |
| Aromatic Amino Acids | F, H, W, Y |
| Tiny Amino Acids | A, C, D, G, S, T |
| Small Amino Acids | E, H, I, L, K, M, N, P, Q, V |
| Large Amino Acids | F, R, W, Y |
| Polar Amino Acids | D, E, R, K, Q, N |
| Charged Amino Acids | D, E, K, H, R |
| Positively Charged Amino Acids | H, K, R |
| Negatively Charged Amino Acids | D, E |

## 1.6 BLOSUM62

BLOSUM62 is a substitution probability matrix that represents the probability of an amino acid being substituted by another amino acid in a protein(7). Each peptide sequence in this study was encoded into a 600-dimensional feature vector. A padding scheme was introduced in peptide sequences shorter than 30 amino acids.

## 1.7 DBAASP physicochemical properties (DBAASP-PP)

The physicochemical properties of ACPs determine their anticancer activity. The DBAASP database was employed to calculate a series of properties, including amphiphilicity index, normalized hydrophobic moment, isoelectric point, normalized hydrophobicity, net charge, penetration depth, propensity to *in vitro* aggregation, tilt angle, linear moment, propensity to disordering, propensity to PPⅡ coil, and angle subtended by the hydrophobic residues*(8)*. The Moon and Fleming scale was used as the hydrophobicity scale for these calculations*(9)*.

## 1.8 eXtreme Gradient Boosting (XGBoost) model

The regulated hyperparameters of the XGBoost model that required optimization are listed in **Table S4**. The model performance was initially evaluated using Matthews Correlation Coefficient (MCC) via 5-fold cross-validation with varying numbers of estimators. A model with 150 estimators achieved an MCC of 0.8188, whereas the model with 280 estimators achieved an MCC of 0.8208. Despite the slight improvement in the latter, we chose the model with 150 estimators to avoid potential overfitting. Subsequent optimization of the remaining parameters was performed using a grid search approach. Based on the MCC results from 5-fold cross-validation across 256 parameter combinations, the optimal values of max_depth, min_child_weight, subsample, and colsample_bytree were 8, 2, 0.7, and 0.7, respectively.

**Table S4.**

Regulated hyperparameters in the XGBoost model.

| **Hyperparameters** | **Value** |
| --- | --- |
| booster | gbtree |
| objective | binary: logistic |
| gamma | 0.1 |
| learning rate | 0.1 |
| reg_alpha | 1 |
| reg_lambda | 1 |
| random_state | 2022 |
| n_estimators | 20、40、60、80、100、110、120、130、140、150、160、170、180、190、200、210、220、230、240、250、260、270、280、290、300、400、405 |
| max_depth | 4、6、8、10 |
| min_child_weight | 2、6、8、10 |
| subsample | 0.5、0.6、0.7、0.8 |
| colsample_bytree | 0.5、0.6、0.7、0.8 |

## 1.9 Deep Neural Network (DNN) model

In the DNN model, four hidden layers were selected to balance predictive performance, overfitting, and computational cost. The parameters considered for optimization are shown in **Table S5**. To further mitigate overfitting, an early stopping strategy was utilized. Different combinations of dropout rates and neuron numbers in the hidden layers were tested, and the configuration yielding the highest MCC in 5-fold cross-validation on the training set was selected. The optimal DNN architecture comprised a dropout rate of 0.2 and a sequence of hidden layers with respective neuron counts of 256, 256, 256, and 128.

**Table S5.**

Parameters for optimization in the Deep Neural Network model.

| **Hyperparameters** | **Value** |
| --- | --- |
| Optimizer | Adam |
| Loss function | BCELoss |
| Dropout | 0.1、0.2、0.3、0.4 |
| Activation function in hidden layers | ReLU |
| Nodes in each hidden layer | 16、32、64、128、256、512 |
| Activation function in the output layer | Sigmoid |

## 1.10 Transfer Learning (TL) model

The network parameters and adjustments in the training DNN model were used to train the pretraining model using AMP sequence data. The optimal parameter combination was determined based on the maximum MCC from the 5-fold cross-validation on the training set, with a dropout rate of 0.2 and the number of neurons in the hidden layers set to 256, 256, 32, and 128, respectively. Subsequently, the TL pretraining model was fine-tuned using the ACP training set to adjust network weights and biases, resulting in the final TL model for anticancer peptide activity prediction.

**Table S6.**

Significance analysis between ACP and non-ACP sequences in terms of the 20 different amino acids at the first position using Fisher’s exact test.

| **Amino acids** | **P value** | **Odds value** | **Amino acids** | **P value** | **Odds value** |
| --- | --- | --- | --- | --- | --- |
| A | 3.34E-11 | 0.3883 | M | 2.90E-172 | 0.0128 |
| C | 5.11E-08 | 6.6066 | N | 0.0191 | 0.5063 |
| D | 0.0019 | 0.4681 | P | 0.3527 | 1.3120 |
| E | 0.0002 | 0.3697 | Q | 0.212 | 0.6794 |
| F | 5.64E-38 | 7.8517 | R | 1.81E-26 | 6.8226 |
| G | 2.96E-20 | 3.5945 | S | 7.36E-09 | 0.3270 |
| H | 0.2241 | 1.6403 | T | 0.0002 | 0.3948 |
| I | 0.0007 | 2.0823 | V | 0.0207 | 0.6401 |
| K | 9.62E-27 | 5.7314 | W | 4.00E-10 | 10.6261 |
| L | 0.0069 | 1.6549 | Y | 0.1107 | 0.5883 |

**Table S7**.

Features of model-predicted oncolytic peptides, including net charge, amphiphilicity index, relative hydrophobicity value, LI value, and proportion of amino acids in helical structure.

| **Peptide sequences** | **Net Charge** | **Amphiphilicity Index** | **Relative Hydrophobicity value** | **LI value** | **Proportion of amino acids in helical structure** |
| --- | --- | --- | --- | --- | --- |
| GLRSKIWLWVLLMIWQESNKFKKM | 4 | 1.68 | 13.24 | 340.5 | 0.92 |
| FLPKLFAKITKKNMAHIR | 5 | 1.03 | 10.78 | 347.4 | 0.89 |
| VWLSALKFIGKHLAKHQLSKL | 4 | 1.23 | 11.06 | 348.8 | 0.86 |
| KRFWQLVPLAIKIYRAWKRR | 7 | 2.05 | 12.45 | 361.3 | 0.85 |
| IWLTALKFLGKNLGKHLAKQQLAKL | 5 | 1.17 | 10.78 | 434.8 | 0.80 |
| KAYSMPRCKYLFRAVLCWL | 4 | 1.54 | 12.48 | 393.4 | 0.74 |
| FLKGCWTKWYSLKPKCPF | 4 | 1.87 | 12.52 | 192.1 | 0.61 |
| RIRFPWPWRWPWWRRVRG | 6 | 2.74 | 15.76 | 91.1 | 0.50 |
| WKKLKKLLKKLKKL | 8 | 2.59 | 10.36 | 242 | 0.43 |
| RIRFPWPWRWPWWPRFRG | 5 | 2.61 | 16.94 | 120.2 | 0.39 |
| KRFGRLAKSFLRMRILLPRRKILLAS | 9 | 0.99 | 11.72 | 472.1 | 0.23 |
| RFGRFLRKIRRFRPKVTITIQGSARF | 9 | 0.99 | 10.5 | 481.1 | 0.23 |
| KSLRPRCWIKIKFRCKSLKF | 8 | 1.63 | 10.97 | 210.8 | 0 |
| RPRCWIKIKFRCKSLKF | 7 | 1.7 | 11.52 | 178.3 | 0 |
| RWCVYAYVRIRGVLVRYRRCW | 6 | 2.08 | 12.39 | 179.5 | 0 |
| VRLLRRRI | 4 | 1.23 | 12.6 | 85.2 | 0 |

# 2. Supplemental results


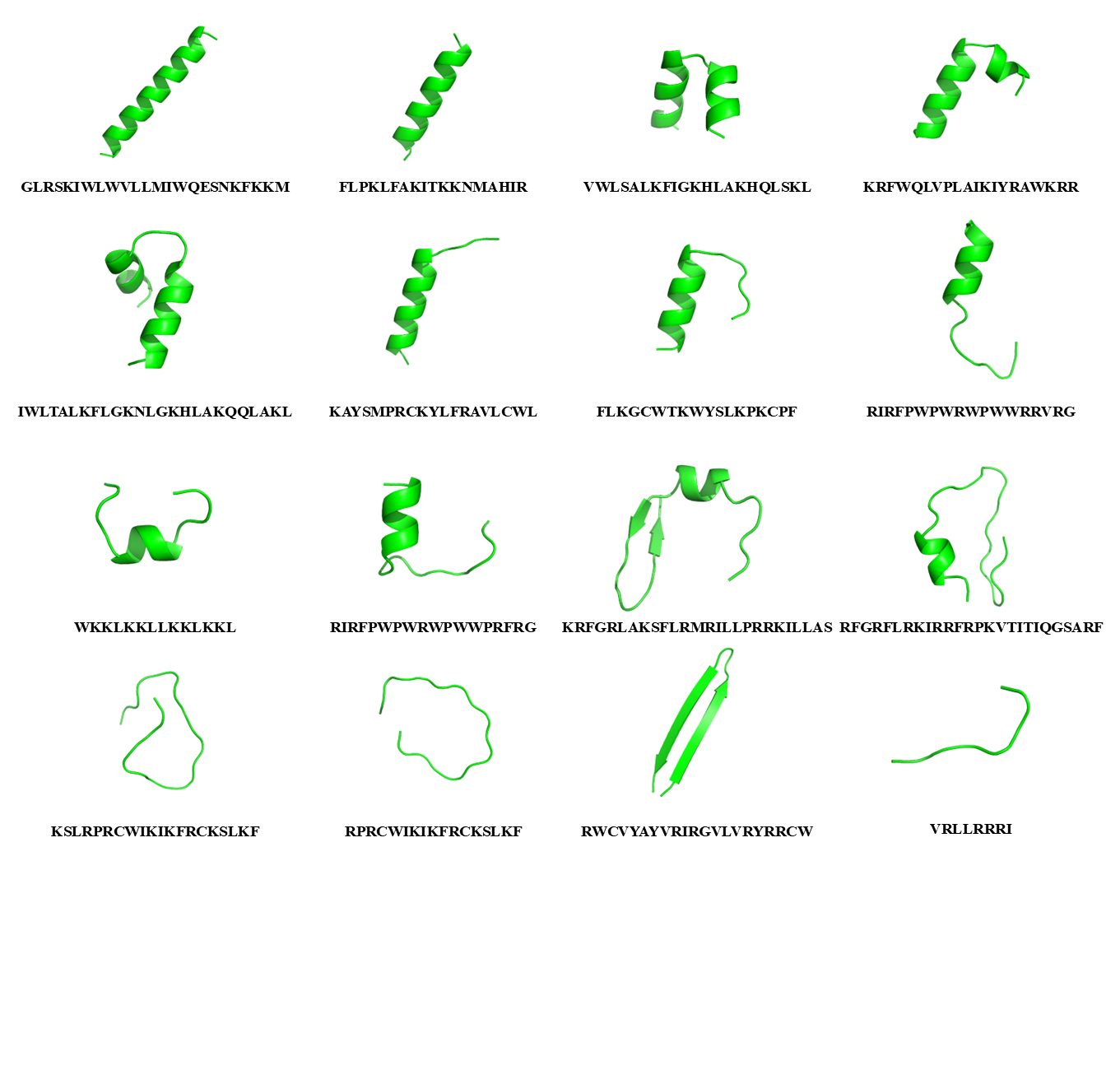


## Figure S1 Predicted structures of the 16 peptides.


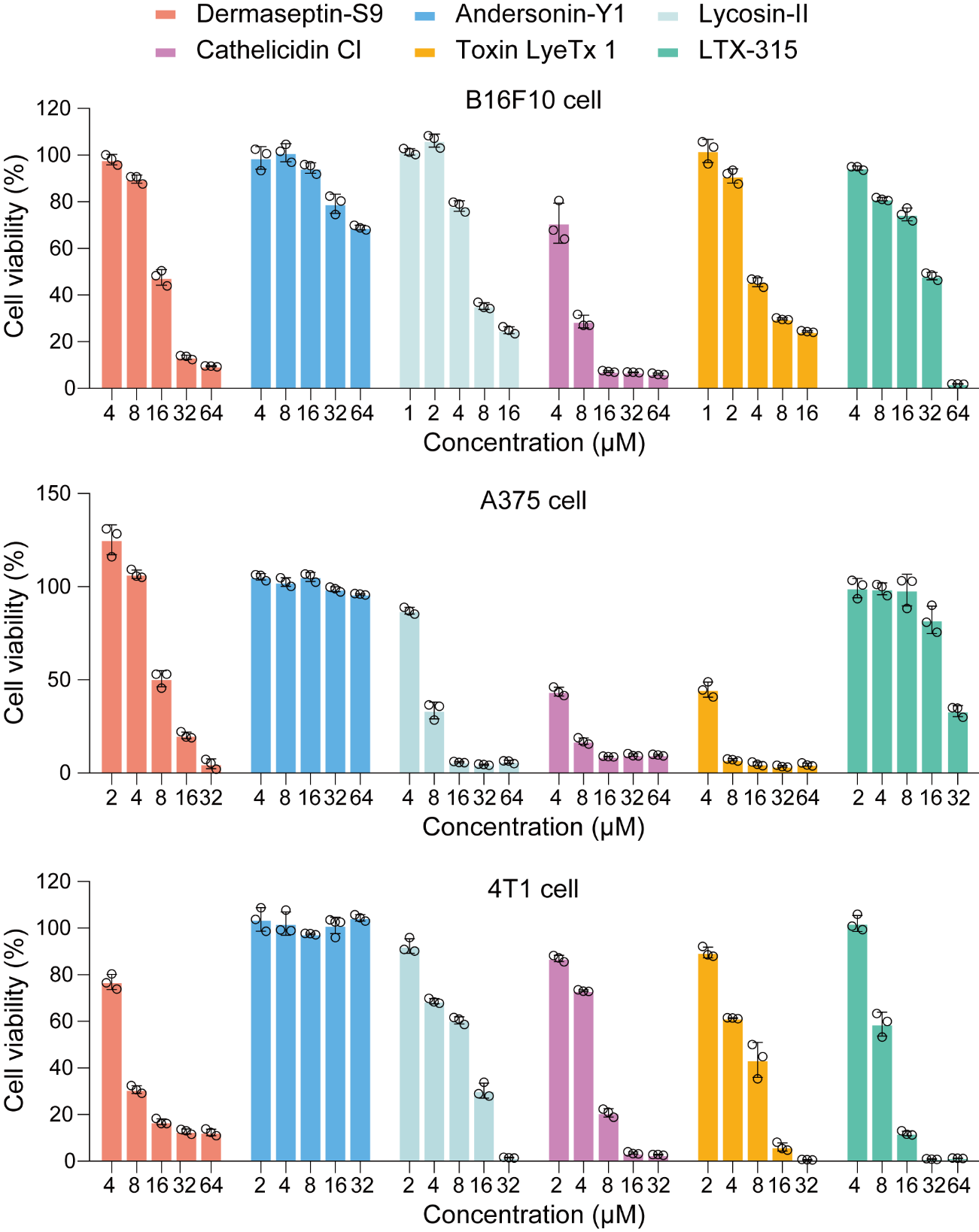


## Figure S2 Cell viabilities of B16F10, A375, and 4T1 cell lines incubated with a series of concentrations of predicted peptides, including Dermaseptin-S9, Andersonin-Y1, Lycosin-II, Cathelicidin CI, Toxin LyeTx 1, and LTX-315.

**
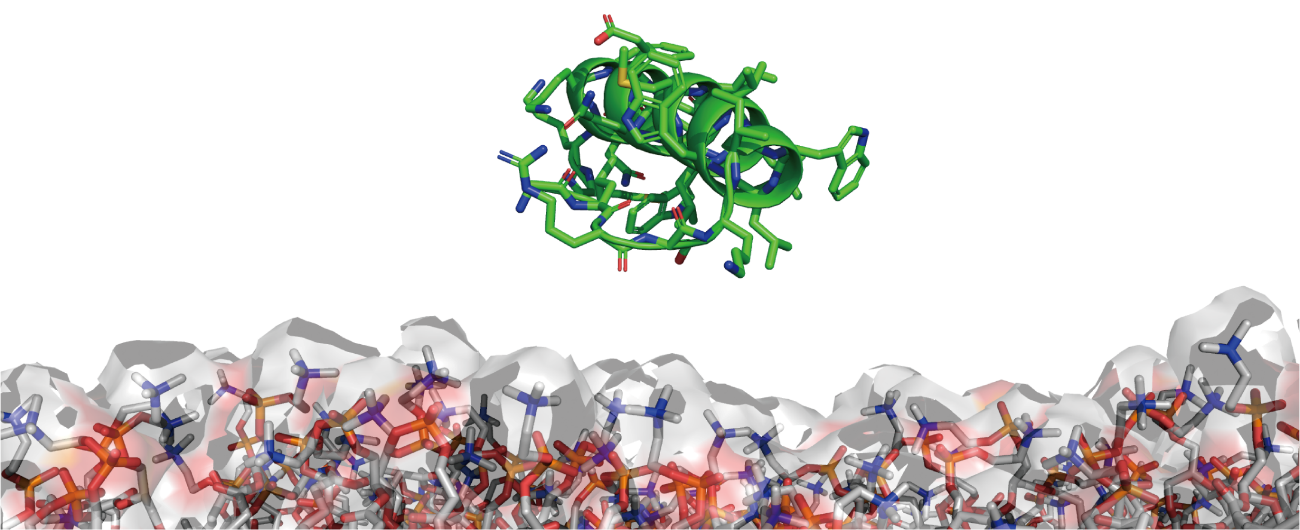
**

## Figure S3 Snapshot showing no interaction between Dermaseptin-S9 and zwitterionic membrane at 500 ns.


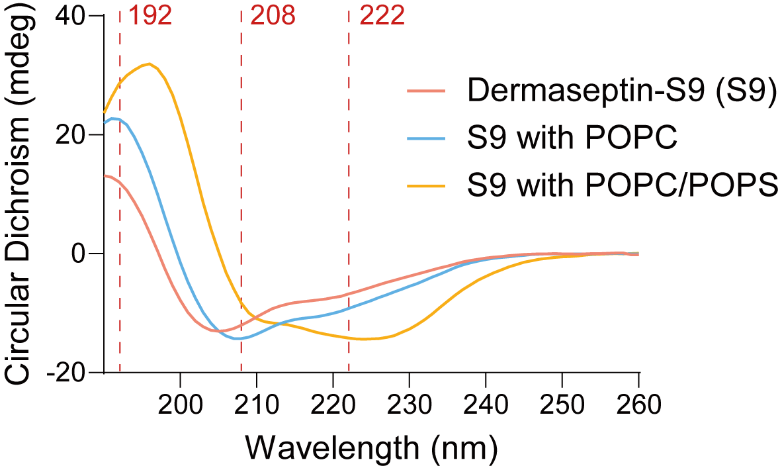


## Figure S4 CD spectra of Dermaseptin-S9 in the absence or presence of POPC or POPC/POPS (4:1) model membranes.


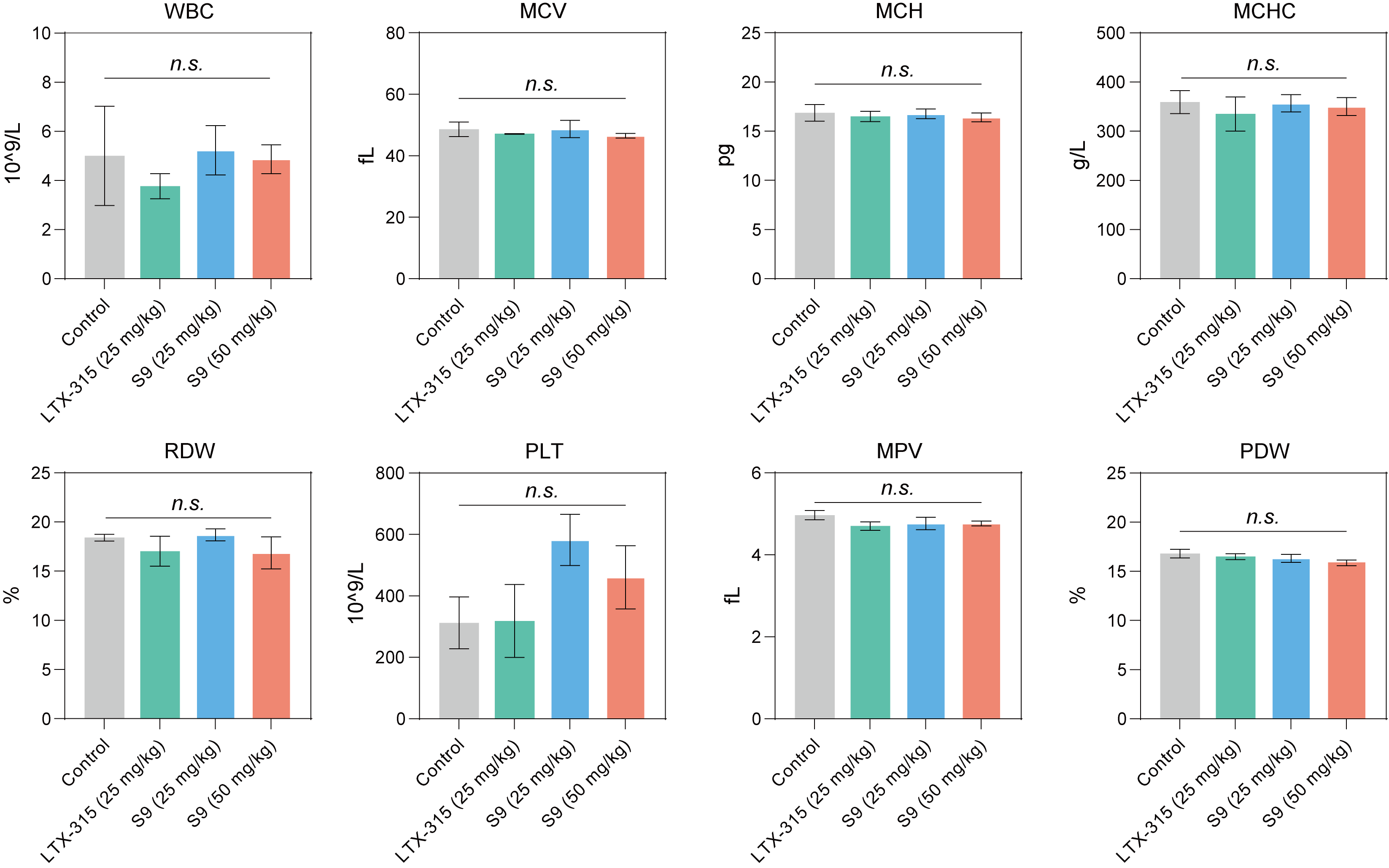
**Figure S5** Blood safety analysis in mice. The blood samples of each group were extracted to assess the safety of the medicine. WBC, white blood cell count; MCV, mean corpuscular volume; MCH, mean corpuscular hemoglobin; MCHC, MHC concentration; RDW, red cell distribution width; PLT, platelet; MPV, mean platelet volume; PDW, platelet distribution width. Data are presented as mean values ± SD (n = 5). n.s., not significant.

**
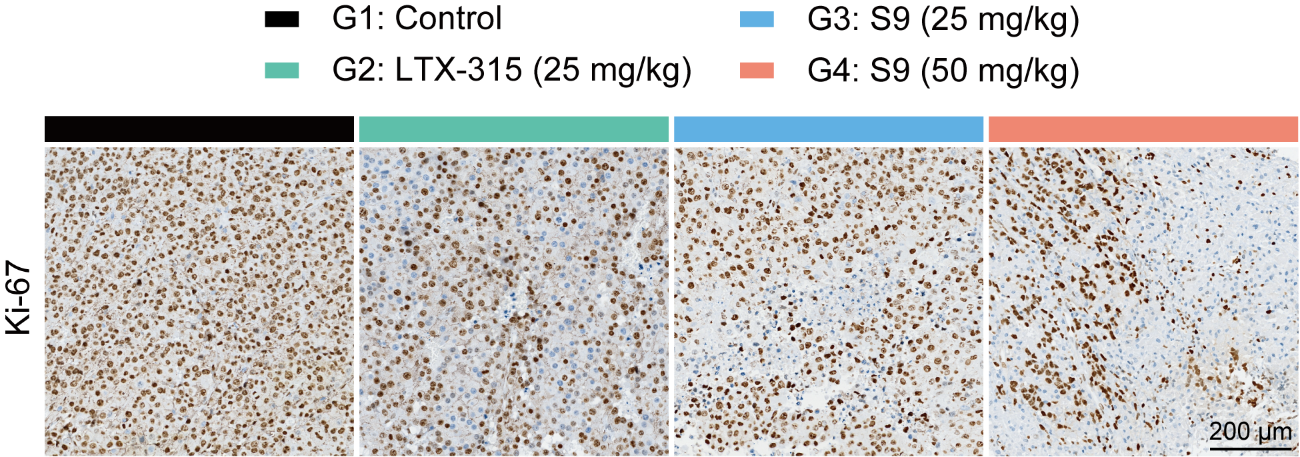
**

## Figure S6 Ki-67 staining images of tumor samples after different treatments.


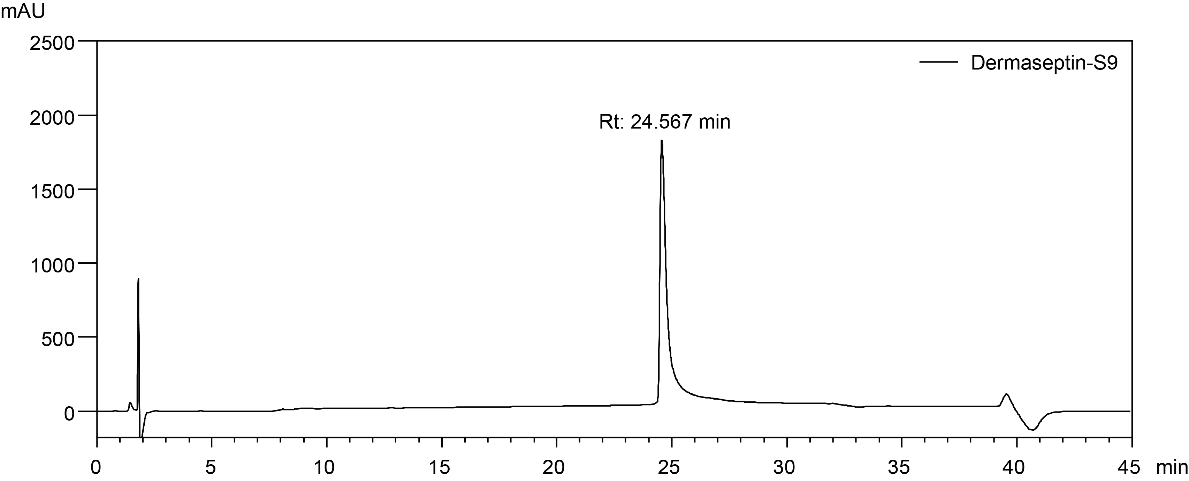


**Figure S7** Analytical HPLC of the peptide Dermaseptin-S9.


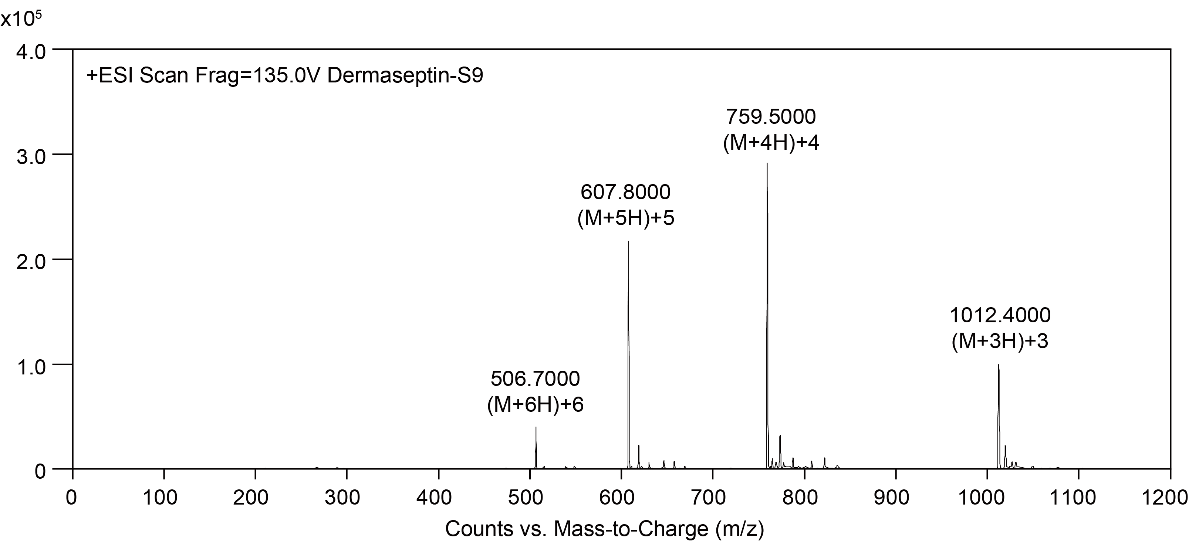


**Figure S8** ESI-MS of the peptide Dermaseptin-S9.


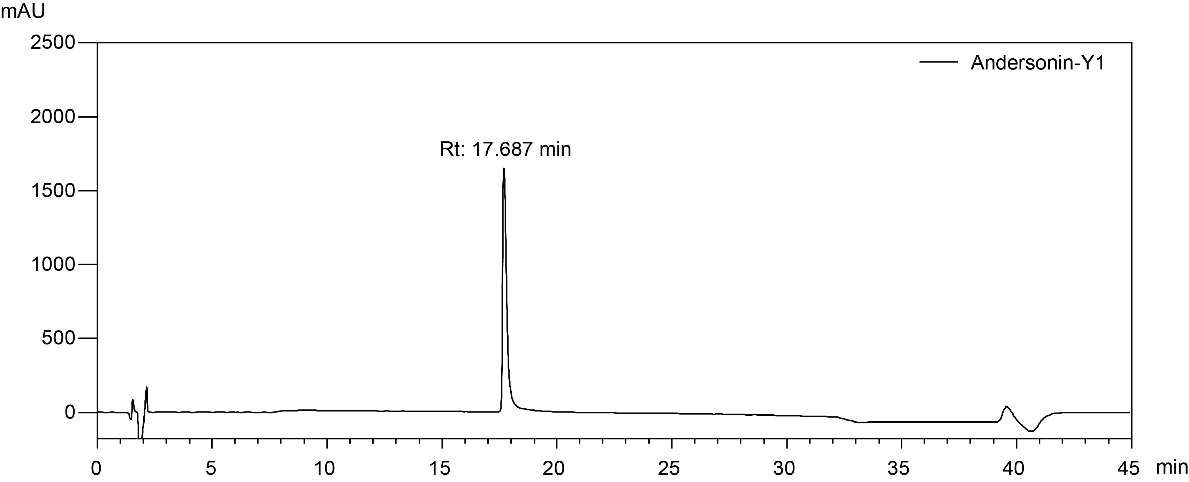


**Figure S9** Analytic HPLC of the peptide Andersonin-Y1.


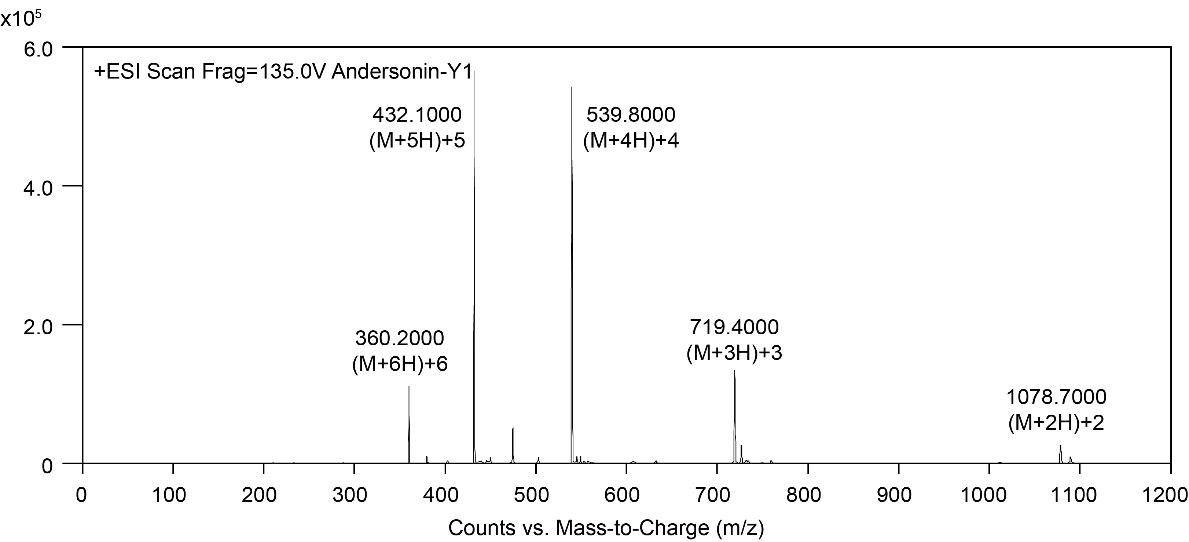


**Figure S10** ESI-MS of the peptide Andersonin-Y1.


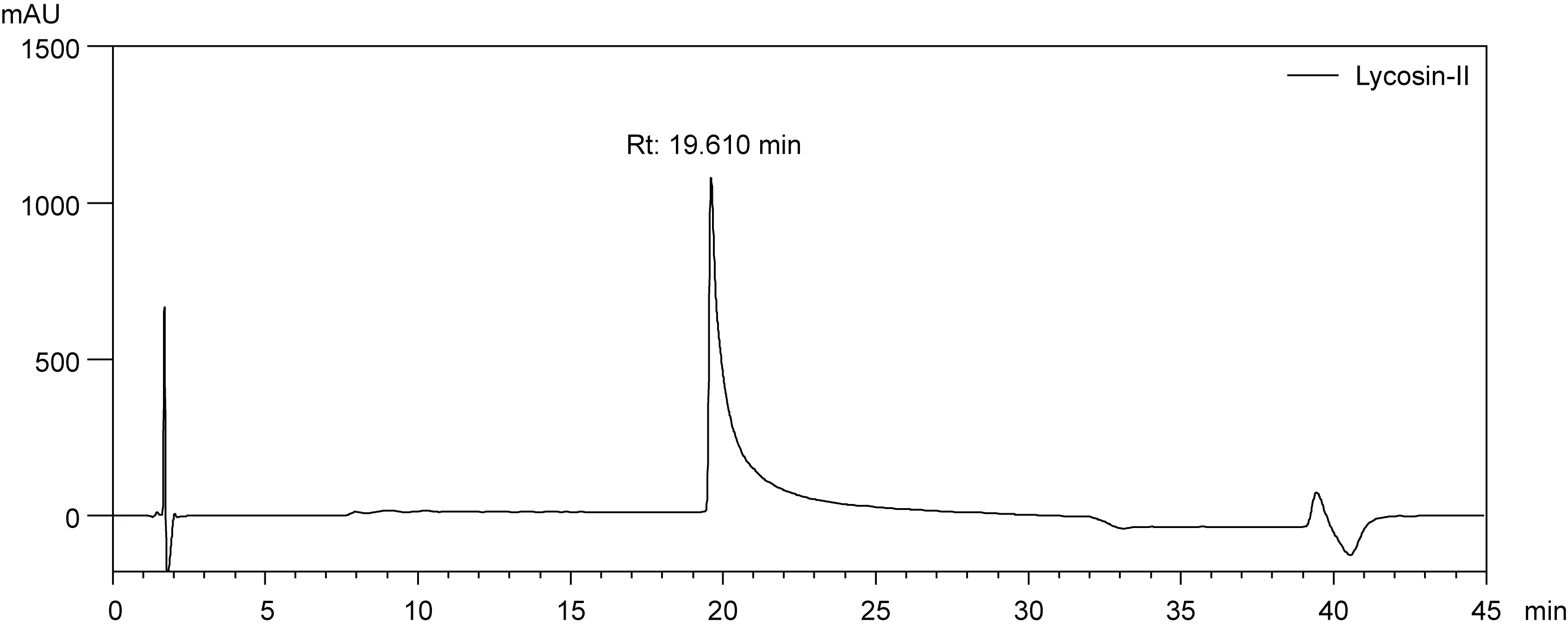


**Figure S11** Analytic HPLC of the peptide Lycosin-II.


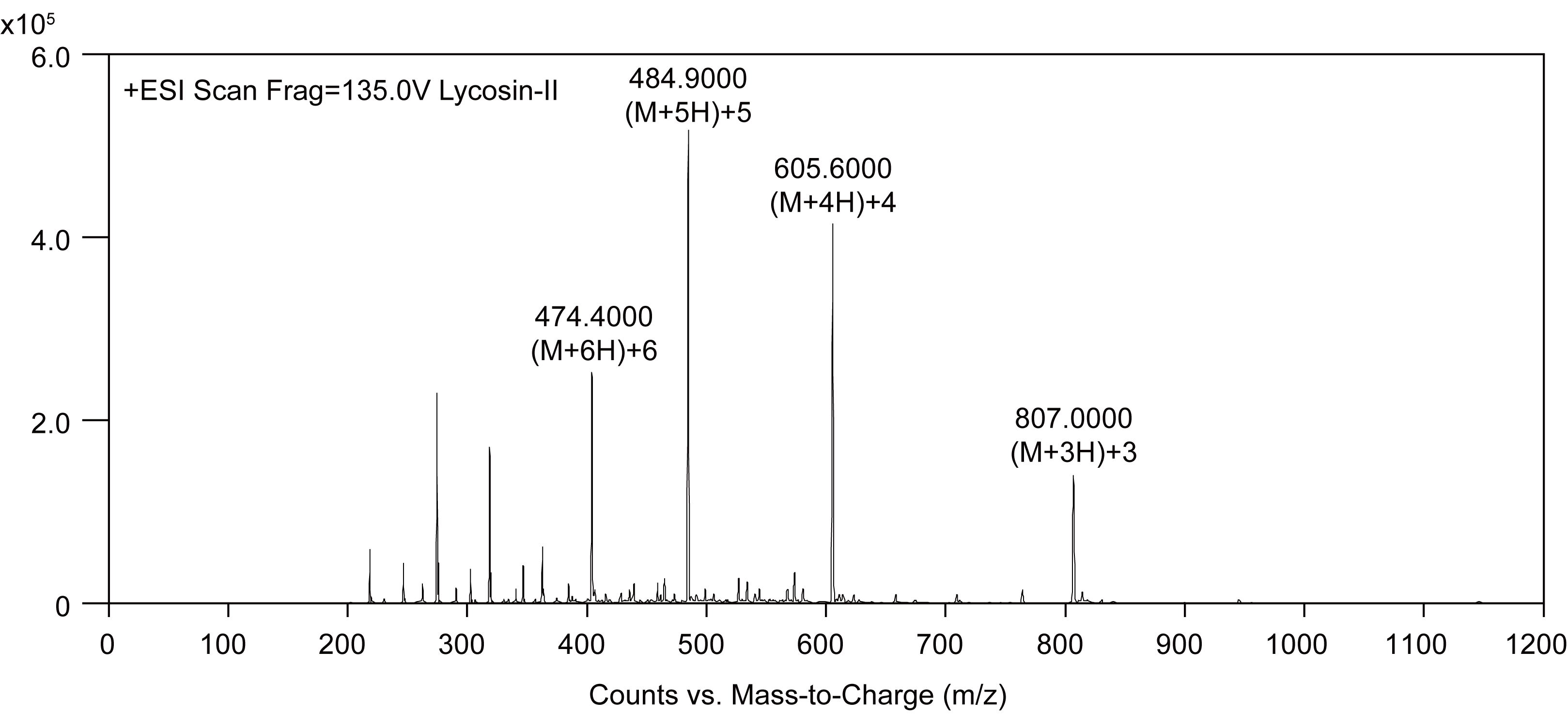


**Figure S12** ESI-MS of the peptide Lycosin-II.


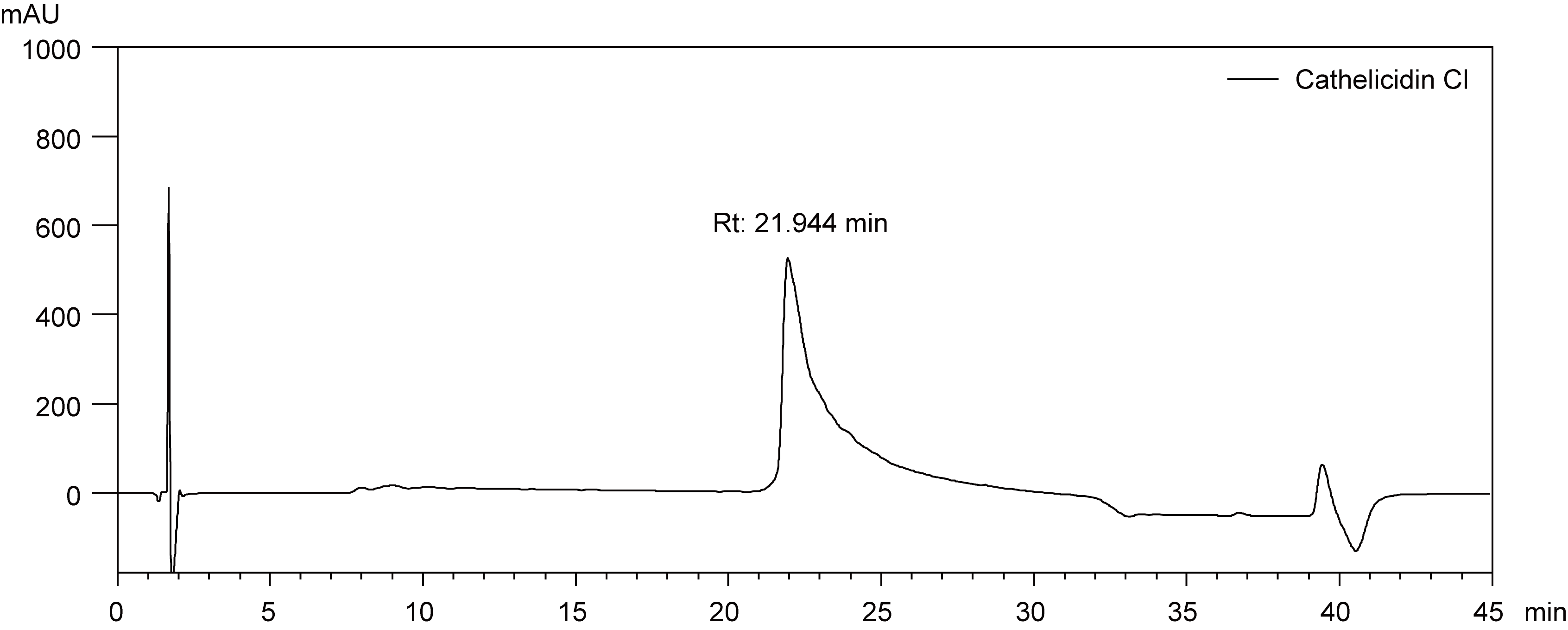


**Figure S13** Analytic HPLC of the peptide Cathelicidin CI.


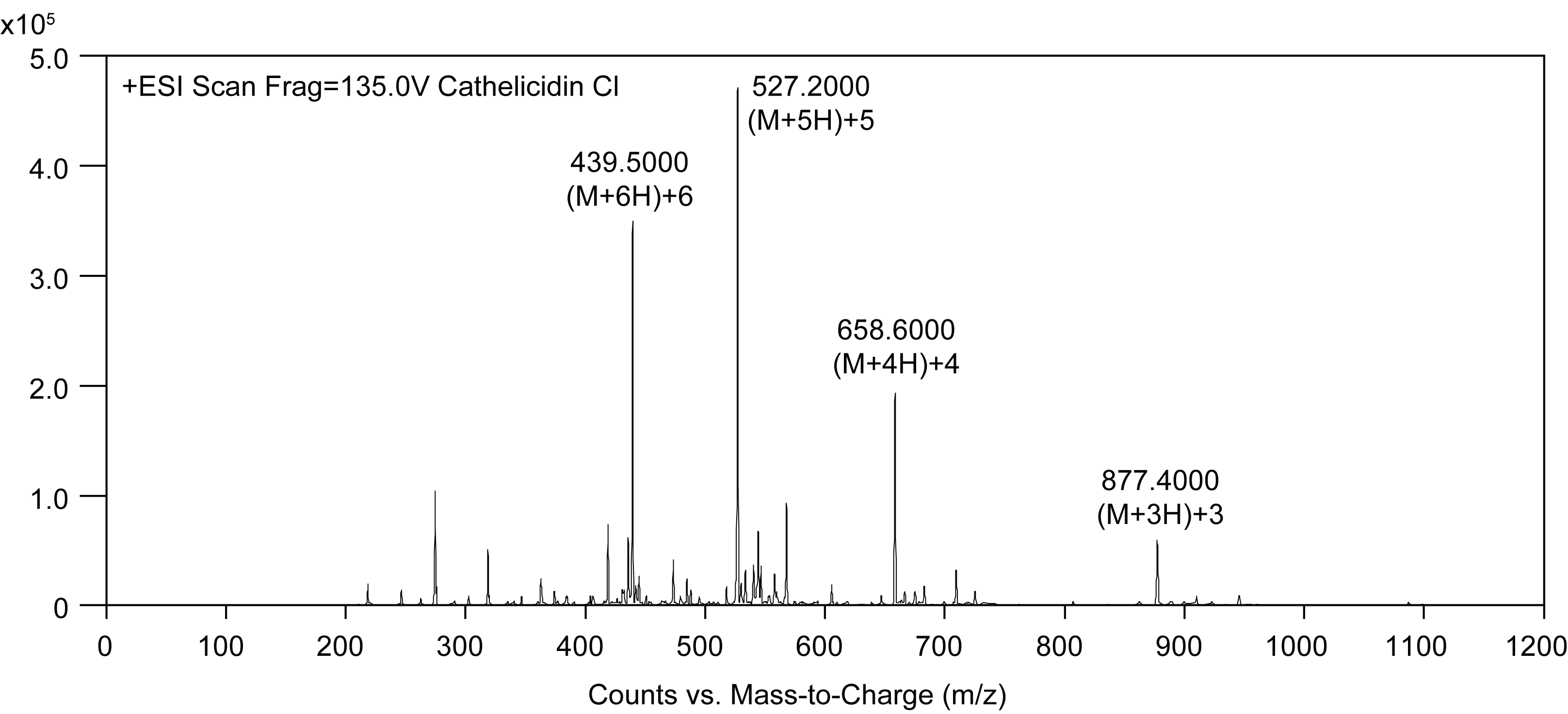


**Figure S14** ESI-MS of the peptide Cathelicidin CI.


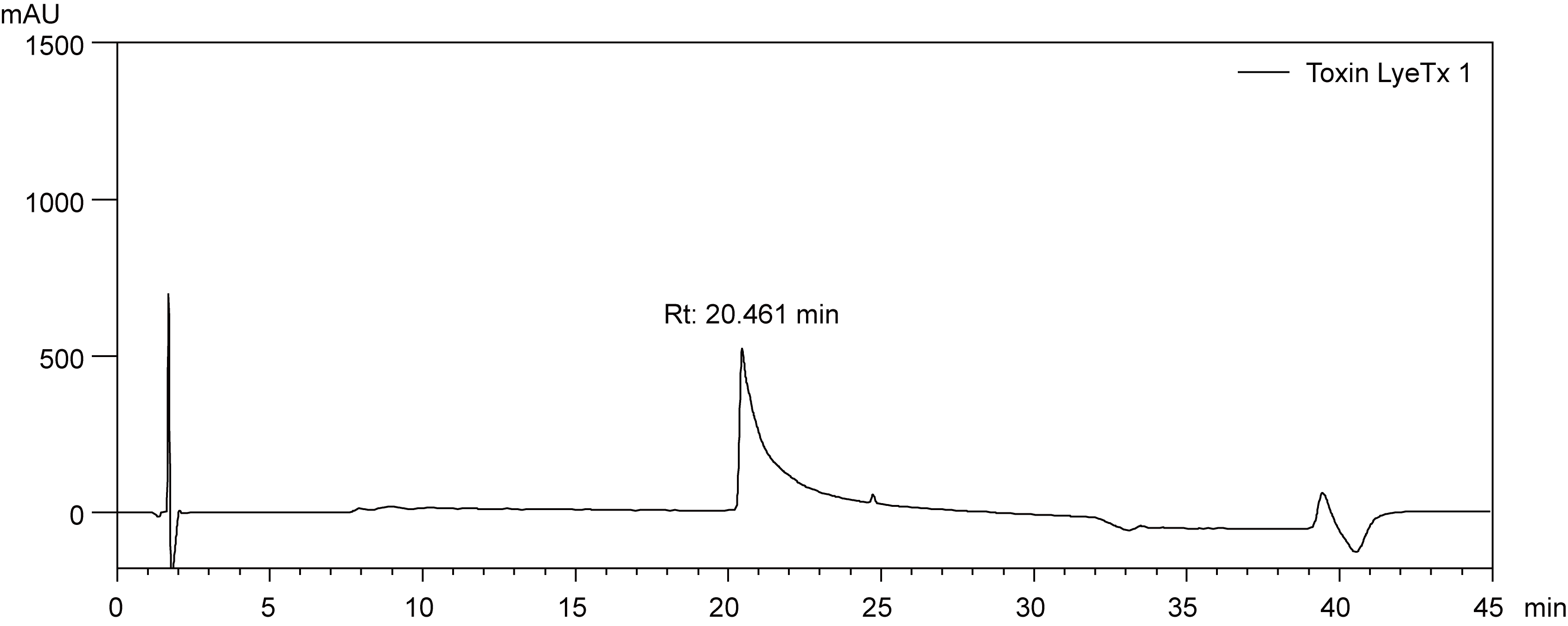


**Figure S15** Analytic HPLC of the peptide Toxin LyeTx 1.


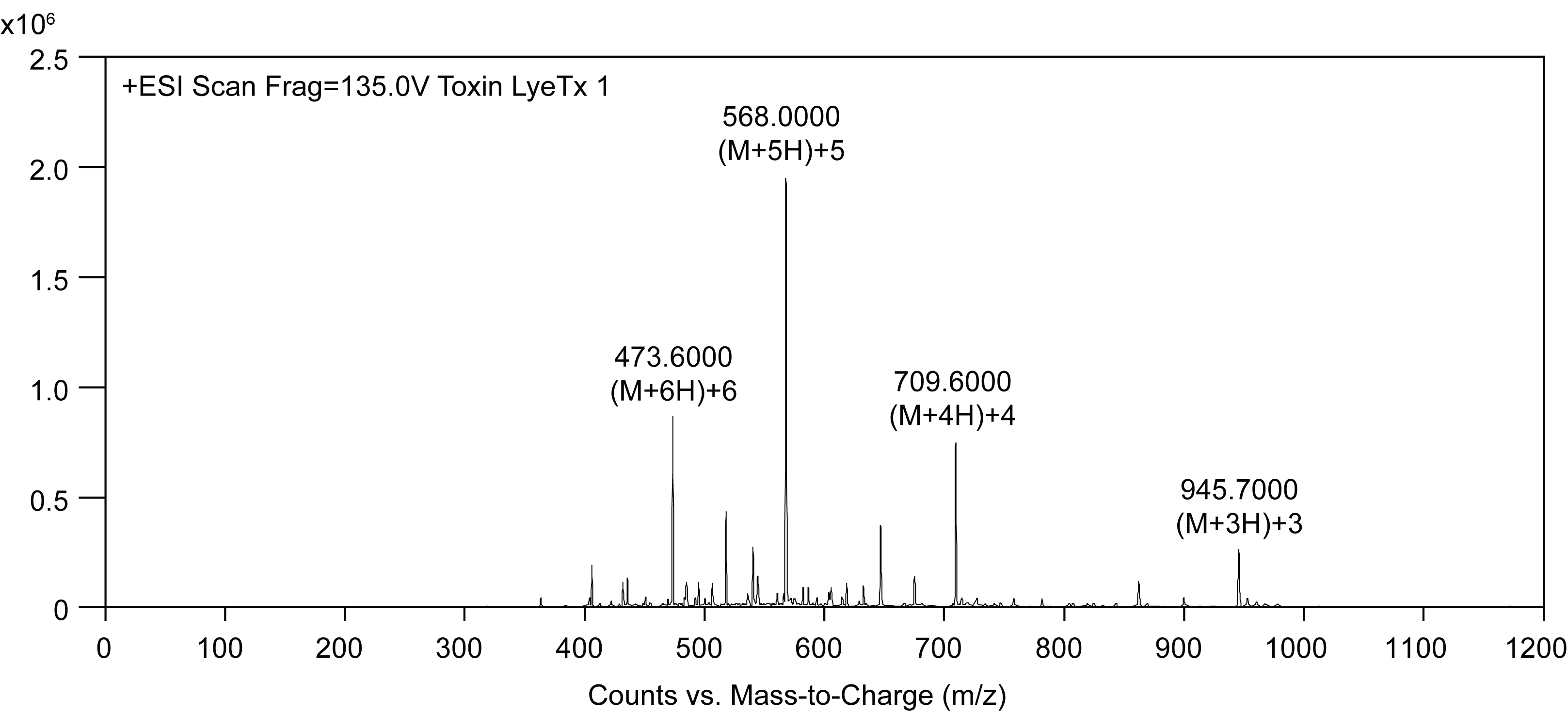


**Figure S16** ESI-MS of the peptide Toxin LyeTx 1.


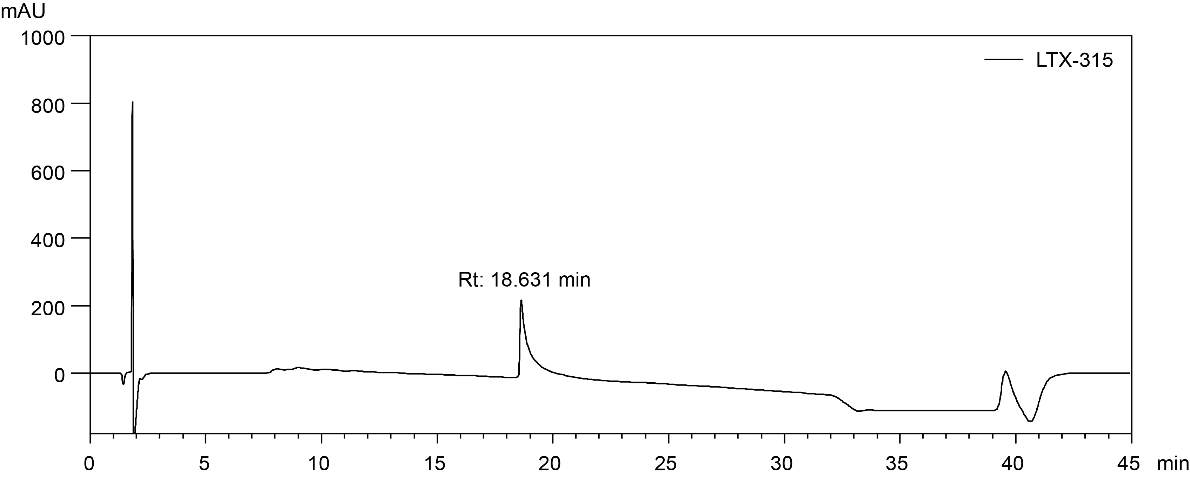


**Figure S17** Analytic HPLC of the peptide LTX-315.


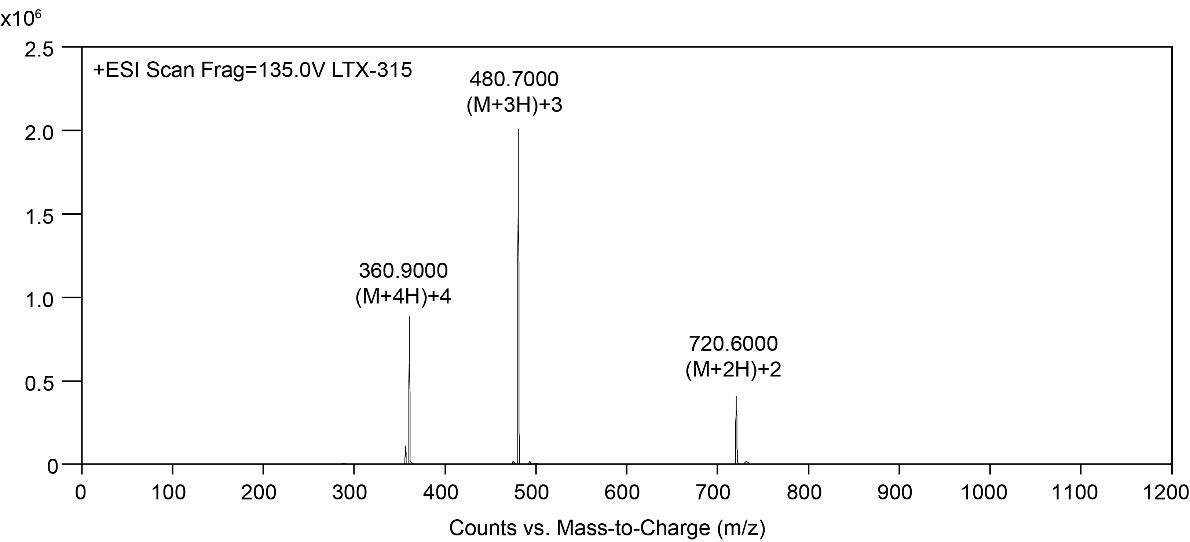


**Figure S18** ESI-MS of the peptide LTX-315.


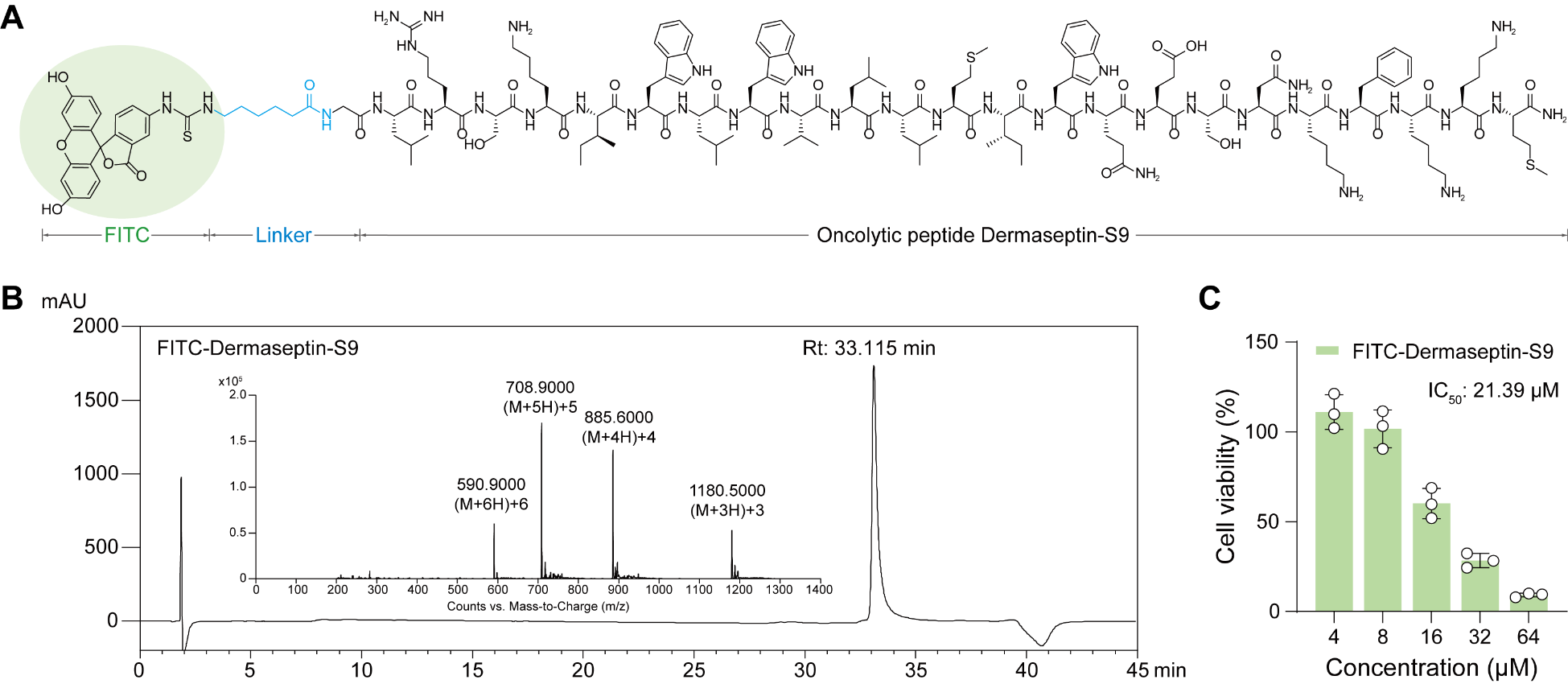


**Figure S19** (A) Molecular structure of FITC-Dermaseptin-S9. (B) HPLC profile and mass spectrum of FITC-Dermaseptin-S9. (C) Antiproliferative activity of FITC-Dermaseptin-S9 in B16F10 cells.

# 3. Supplementary References

1. G. Govindan, A. S. Nair, "Composition, Transition and Distribution (CTD) — A dynamic feature for predictions based on hierarchical structure of cellular sorting," *2011 Annual IEEE India Conference* **2011**, 1-6.

2. R. Kumar, K. Chaudhary, J. Singh Chauhan et al., "An in silico platform for predicting, screening and designing of antihypertensive peptides," *Sci Rep* **2015**, 5(1), 12512. <https://doi.org/10.1038/srep12512>

3. F. M. Li, X. Q. Wang, "Identifying anticancer peptides by using improved hybrid compositions," *Sci Rep* **2016**, 6, 33910. <https://doi.org/10.1038/srep33910>

4. Y.L. Chen, Q.Z. Li, "Prediction of the subcellular location of apoptosis proteins," *Journal of Theoretical Biology* **2007**, 245(4), 775-783. [https://doi.org/10.1016/j.jtbi.2006.11.010](https://doi.org/https:/doi.org/10.1016/j.jtbi.2006.11.010)

5. J. Pánek, I. Eidhammer, R. Aasland, "A new method for identification of protein (sub)families in a set of proteins based on hydropathy distribution in proteins," *Proteins* **2005**, 58(4), 923-934. <https://doi.org/10.1002/prot.20356>

6. P. Feng, Z. Wang, "Recent advances in computational methods for identifying anticancer peptides," *Curr Drug Targets* **2019**, 20(5), 481-487. <https://doi.org/10.2174/1389450119666180801121548>

7. S. Ahmed, R. Muhammod, Z. H. Khan et al., "ACP-MHCNN: an accurate multi-headed deep-convolutional neural network to predict anticancer peptides," *Sci Rep-Uk* **2021**, 11(1), 23676. <https://doi.org/10.1038/s41598-021-02703-3>

8. M. Pirtskhalava, A. A. Amstrong, M. Grigolava et al., "DBAASP v3: database of antimicrobial/cytotoxic activity and structure of peptides as a resource for development of new therapeutics," *Nucleic Acids Res* **2021**, 49(D1), D288-D297. <https://doi.org/10.1093/nar/gkaa991>

9. C. P. Moon, K. G. Fleming, "Side-chain hydrophobicity scale derived from transmembrane protein folding into lipid bilayers," *Proc Natl Acad Sci* **2011**, 108(25), 10174-10177. <https://doi.org/10.1073/pnas.1103979108>
